# Development and Validation of a UPLC-MS/MS Method for the Determination of Emerging Synthetic, Semi-Synthetic and Related Cannabinoids in Human Hair

**DOI:** 10.3390/molecules31213816

**Published:** 2026-10-22

**Authors:** Leonardo Romani, Giulio Mannocchi, Lucrezia Stefani, Luigi Tonino Marsella, Roberta Tittarelli

**Affiliations:** Department of Biomedicine and Prevention, University of Rome “Tor Vergata”, Via Montpellier, 1, 00133 Rome, Italy; leonardo.romani@uniroma2.it (L.R.); giulio.mannocchi@uniroma2.it (G.M.); l.stefani@med.uniroma2.it (L.S.); marsella@uniroma2.it (L.T.M.)

**Keywords:** UPLC-MS/MS, forensic toxicology, synthetic cannabinoids, hair analysis, method validation

## Abstract

Synthetic cannabinoids (SCs) are an evolving group of new psychoactive substances (NPSs), characterized by structural diversity and continuously emerging analogues. Their detection in biological matrices is challenging because of low concentrations, rapid metabolism, and incomplete coverage by routine methods. Hair analysis offers a detection window for retrospective exposure assessment. This study aimed to develop and validate an ultra-performance liquid chromatography–tandem mass spectrometry (UPLC–MS/MS) method for simultaneous identification and quantification of 26 synthetic, semi-synthetic, and related cannabinoids in human hair and to assess its applicability to authentic forensic samples. The method was validated for linearity, limits of detection (LOD) and quantification (LLOQ), accuracy, precision, matrix effect, recovery and process efficiency, with additional robustness assessments under freeze–thaw and processed-extract conditions, and was then applied to *n* = 100 hair samples collected from subjects tested positive for Δ^9^-tetrahydrocannabinol (Δ^9^-THC) during forensic assessments following driving license suspension. LODs were 1.56–3.13 pg/mg, LLOQs 6.25–12.5 pg/mg, and R^2^ ≥ 0.992; accuracy and precision met the predefined acceptance criteria, while ME, RE, and PE showed generally consistent mean performance with wider level-specific variability for selected analytes. Target cannabinoids were detected in 23/100 samples, with 14 different compounds identified. MDMB-4en-PINACA, 9R-HHC-O, and ADB-BUTINACA were the most frequently detected compounds. The method enabled sensitive and reliable detection of multiple emerging cannabinoid classes in hair, supporting the continuous updating of targeted analytical panels in response to the evolving NPS landscape.

## 1. Introduction

Synthetic cannabinoids (SCs) represent a highly dynamic class of new psychoactive substances (NPSs) and pose a significant challenge to forensic toxicology. Originally developed for pharmacological research, these compounds rapidly emerged on the illicit drug market as cannabis substitutes and have continued to evolve through the introduction of new analogues designed to circumvent legal controls and routine analytical detection [1,2,3,4].

The toxicological relevance of SCs is primarily related to their high potency at cannabinoid receptors, resulting in severe neurological, cardiovascular, and psychiatric adverse effects [5,6,7,8,9,10,11]. According to the European Union Drugs Agency (EUDA), 20 new cannabinoids were identified in Europe in 2024, 18 of which were semi-synthetic cannabinoids, accounting for more than 40% of all substances newly reported to the EU Early Warning System [12]. These findings highlight the need for continuous analytical surveillance and for methods capable of detecting an evolving range of cannabinoids [3,4,12].

The most prevalent subclasses of SCs include indole- and indazole-based derivatives such as PINACA, FUBINACA, CHMINACA, CUMYL, MDMB, BUTINACA, and the more recently identified OXIZID compounds [3,13,14]. The illicit market has also experienced a rapid expansion of semi-synthetic cannabinoids, including hexahydrocannabinol (HHC), hexahydrocannabiphorol (HHC-P), tetrahydrocannabiphorol (THCP), hexahydrocannabihexol (HHC-H), and related analogues derived from hemp cannabinoids [12].

The detection of these compounds remains particularly challenging due to extensive metabolism, low concentrations in biological specimens, and the continuous emergence of novel substances that may not be included in routine screening methods [13,15]. While conventional biological matrices such as blood and urine are primarily informative of recent drug consumption, hair provides a longer detection window and allows retrospective assessment of drug exposure over several months [16,17,18]. SCs can frequently be detected in hair as unchanged parent compounds, facilitating the identification of newly emerging substances for which metabolic data may be limited or unavailable [14,17,19,20,21,22,23]. Consequently, hair has become an important matrix in forensic and medico-legal investigations, including the assessment of chronic exposure, abstinence monitoring, and driving license regranting procedures [16,17,18,19,24].

Despite these advantages, synthetic and semi-synthetic cannabinoids are generally present in hair at very low concentrations, and matrix effects may significantly affect analytical performance [14,17,20,21,22,23,25]. Liquid chromatography coupled with tandem mass spectrometry (LC-MS/MS) is the analytical technique of choice for their determination because of its high sensitivity, selectivity, and suitability for multi-analyte determination in complex biological matrices [25,26,27]. Published LC-MS/MS methods have demonstrated the feasibility of sensitive multi-analyte cannabinoid determination in hair; however, they differ substantially in analytical scope, target composition, sample preparation, and intended forensic application [12,14,20,21,22,23].

Several studies have reported the validation of UPLC-MS/MS methods for the determination of synthetic cannabinoids in hair samples. Yang et al. (2024), Kutzler et al. (2024), and Liying et al. (2022) [14,19,22] described analytical approaches targeting different panels, generally based on hair washing, predominantly methanolic extraction, and tandem mass spectrometric analysis. However, these methods still present several limitations, including non-harmonized sample preparation protocols, different sample amounts, diverse extraction procedures and analytical panels that may rapidly become outdated because of the continuous emergence of new synthetic and semi-synthetic cannabinoid analogues. Therefore, despite the established suitability of UPLC-MS/MS for this purpose, there remains a need for more standardized, rapid, and updatable analytical methods for the analysis of synthetic cannabinoids in human hair.

The aim of our study was to develop and validate a targeted UPLC–MS/MS method for the simultaneous determination of a panel of emerging synthetic and semi-synthetic cannabinoids in human hair, in accordance with international guidelines for forensic toxicology validation [28,29]. The validated method was subsequently applied to authentic hair samples collected from individuals undergoing forensic toxicological analyses following license suspension for driving under the influence (DUI) of alcohol and/or drugs at the Forensic Toxicology Laboratory of the University of Rome Tor Vergata, demonstrating its applicability in routine forensic casework.

## 2. Results

The developed UPLC-MS/MS method targeted 26 synthetic, semi-synthetic, and related cannabinoids in hair and was validated before being applied to 100 authentic forensic samples; the complete analytical workflow and experimental conditions are described in Section 4.

### 2.1. Method Validation

The analytical method was validated according to established forensic toxicology validation guidelines [28,29]. The validation protocol included assessment of selectivity and specificity, limits of detection (LOD) and quantification (LLOQ), linearity, accuracy, precision, matrix effect, recovery, process efficiency, and carry-over, together with additional robustness assessments under freeze–thaw and processed-extract conditions.

#### 2.1.1. Selectivity and Specificity

Selectivity and specificity were evaluated to assess the reliability of analyte identification in hair samples and to exclude the possible presence of chromatographic or matrix-related interferences.

Blank hair obtained from a single donor and previously confirmed to be free of drugs of abuse and psychoactive substances was used for the selectivity assessment. The retention times were established under the optimized chromatographic conditions, with each experiment performed in triplicate, and each sample injected three times.

Selectivity was assessed using blank hair samples fortified with the selected internal standards to verify the absence of interfering signals at the retention times of the target analytes. Blank samples were then fortified with the representative cannabinoids, including Δ^9^-tetrahydrocannabinol (Δ^9^-THC), cannabidiol (CBD), and cannabinol (CBN). Additional substances commonly encountered in forensic toxicology, including cocaine, benzoylecgonine, opioids, methadone, and amphetamine-type compounds, were also tested to evaluate potential chromatographic interferences.

**Figure 1:**
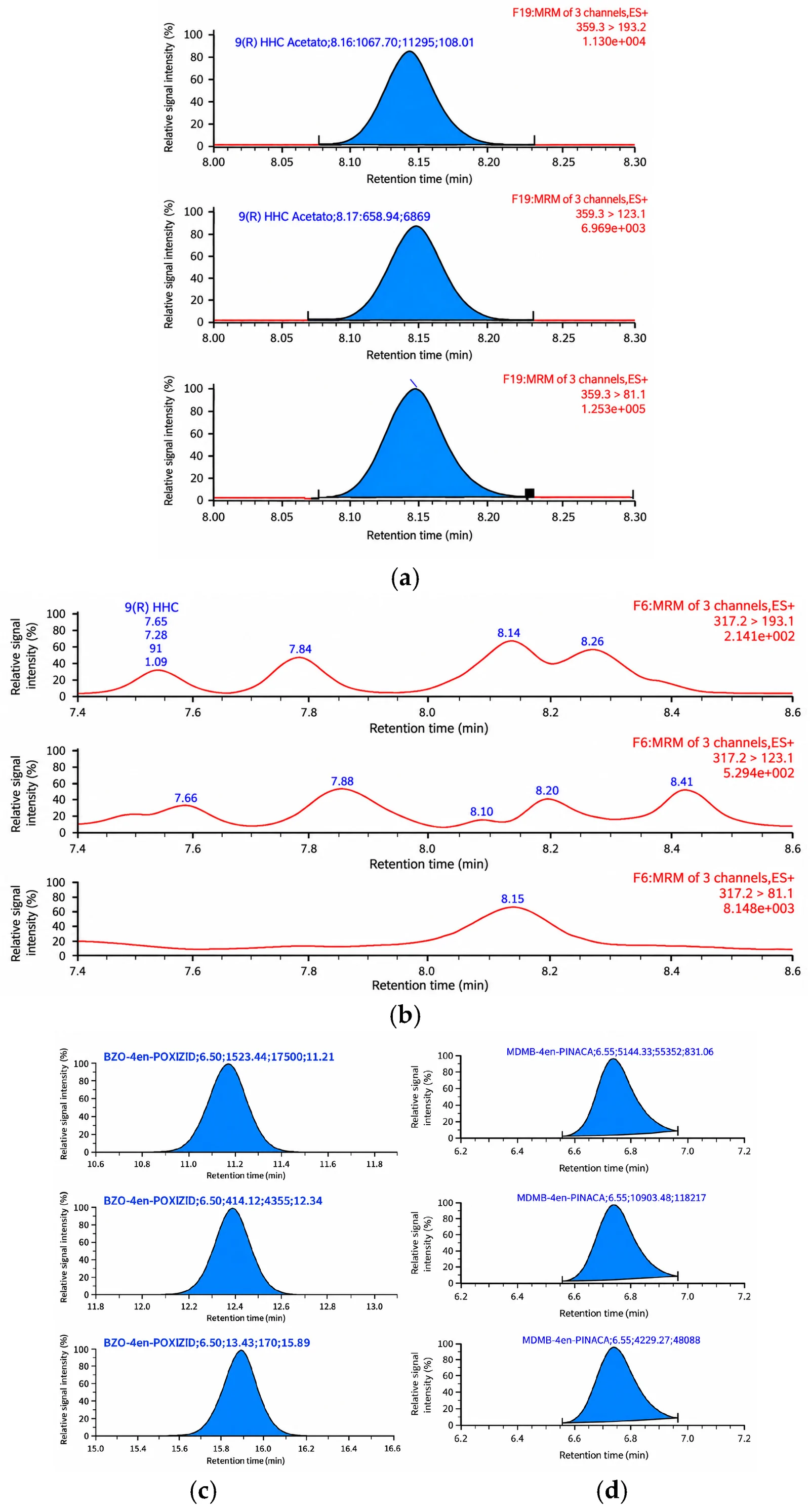
Representative MRM chromatograms illustrating selected aspects of method selectivity and chromatographic performance in hair matrix: (**a**) 9R-HHC-O MRM channel and (**b**) 9R-HHC MRM channel from the same blank hair sample fortified exclusively with 9R-HHC-O at the high QC level (300 pg/mg) and subjected to the complete M3 treatment at 100 °C for 60 min; the signal observed in the 9R-HHC channel remained below the validated 9R-HHC LOD (3.13 pg/mg); (**c**) blank hair fortified with BZO-4en-POXIZID at its analyte-specific LLOQ (6.25 pg/mg); (**d**) an authentic positive hair sample containing MDMB-4en-PINACA (observed RT 6.55 min; reference RT 6.50 min). Retention times shown in the chromatograms correspond to the individual injections all observed deviations were within the predefined ±2% acceptance window.

No interfering peaks were observed in blank hair samples at the retention times of the target analytes or internal standards. In particular, no interfering signals were observed for BZO-4en-POXIZID or BZO-POXIZID in the presence of cocaine and benzoylecgonine. Because of the potential susceptibility of acetate-containing cannabinoids to hydrolysis, the individually fortified samples were also reviewed for evidence of cross-analyte formation during sample preparation. Blank hair fortified exclusively with 9R-HHC-O at the high QC level (300 pg/mg) was subjected to the complete M3 treatment at 100 °C for 60 min. The corresponding 9R-HHC-O and 9R-HHC MRM chromatograms are shown in Figure 1a,b. In the 9R-HHC MRM channel, the response observed in the 317.2 → 81.1 transition at approximately 8.15 min was chromatographically coincident with the 9R-HHC-O peak rather than with the 9R-HHC retention time. This observation is consistent with in-source fragmentation of 9R-HHC-O rather than with formation of 9R-HHC by deacetylation during sample preparation. A separate low-intensity response was observed at 7.65 min, close to the expected 9R-HHC retention time; however, its S/N was 1.09, below the predefined S/N ≥ 3 detection criterion. Therefore, no quantitatively reportable 9R-HHC formation was observed. Using the validated LOD of 3.13 pg/mg as a conservative upper bound for possible 9R-HHC formation, the corresponding molar conversion of the initially added 9R-HHC-O to 9R-HHC was <1.2%. In Figure 1b, the three traces correspond to the 9R-HHC MRM transitions 317.2 → 193.1, 317.2 → 123.1, and 317.2 → 81.1; minor signals not meeting the predefined identification criteria were considered non-specific/background responses. These findings do not support substantial deacetylation of 9R-HHC-O to 9R-HHC under the applied sample-preparation conditions. However, a dedicated paired t = 0 versus t = 60 min stability experiment was not included in the original validation protocol. All analytes showed stable retention times and reproducible MRM transitions under the applied analytical conditions. The retention times were established under the optimized method conditions, whereas the values shown in Figure 1 correspond to the retention times observed in the individual representative injections. The observed differences were within the predefined ±2% retention-time acceptance window. Representative chromatograms illustrating chromatographic performance in the hair matrix are shown in Figure 1, including blank hair fortified with BZO-4en-POXIZID at its analyte-specific LLOQ (6.25 pg/mg), and an authentic positive hair sample containing MDMB-4en-PINACA at an observed retention time of 6.55 min. Δ^9^-THC-P and Δ^8^-THC-P were characterized individually in separate injections, with retention times of 7.92 and 7.98 min, respectively. Because a co-injection of the two isomers was not performed as part of the original validation protocol, their chromatographic resolution under simultaneous conditions was not experimentally established and an Rs value cannot be reported. Accordingly, the retention times obtained from the separate injections should not be interpreted as evidence of chromatographic resolution between the two isomers. Neither Δ^9^-THC-P nor Δ^8^-THC-P was detected in any of the authentic samples analyzed in the present study. Particular attention was also given to the chromatographic distinction between Δ^8^-THC and Δ^9^-THC because the two compounds share the monitored transitions *m*/*z* 315.2 → 193.1 and 315.2 → 123.0 and their ±2% retention-time acceptance windows overlap. In the authentic sample containing Δ^8^-THC, two distinct chromatographic peaks were observed within the same injection in both monitored transitions, at retention times of 7.35 and 7.50 min. The earlier peak corresponded to Δ^9^-THC, whereas the later peak was consistent with the Δ^8^-THC reference-standard retention time of 7.49 min, representing a deviation of only 0.01 min (approximately 0.13%). The Δ^9^-THC concentration in this sample was 0.44 ng/mg. The observation of two discrete peaks in the same injection, together with the agreement of the later peak with the Δ^8^-THC reference retention time and its presence in both monitored transitions, supported the assignment of the later peak to Δ^8^-THC. No relevant chromatographic or matrix-related interferences were observed for the analytes assessed under the selectivity conditions described above. The presence of Δ^9^-THC, CBD, CBN, cocaine and its metabolites, opioids, methadone, and amphetamine-type compounds did not produce significant signal alterations, chromatographic co-elution, or interfering fragmentation patterns. No relevant background interference was observed. Carry-over was assessed by injecting a blank immediately after the highest matrix-matched calibrator (400 pg/mg); no relevant target-analyte signal was observed in the subsequent blank injection. During authentic-sample analysis, an additional blank was injected immediately after any sample producing an analytical response above the validated ULOQ. Periodic blank injections were also included throughout the analytical sequence to monitor potential carry-over. No relevant target-analyte signals were observed in these blank injections. Potential contribution of the M + 2 isotopologue of native Δ^9^-THC to the 9R-HHC MRM channel was specifically assessed using blank hair fortified with Δ^9^-THC alone at 7 ng/mg and subjected to the complete sample-preparation procedure. No signal was observed in the 9R-HHC MRM channel at the 9R-HHC retention time of 7.57 min. Because the tested Δ^9^-THC concentration was slightly higher than the maximum concentration observed in the authentic sample set (6.38 ng/mg), no relevant contribution of the Δ^9^-THC M + 2 isotopologue to 9R-HHC identification was observed over the concentration range encountered in the authentic samples. In the same Δ^9^-THC-only experiment, in which Δ^9^-THC-d3 was not added, the signal observed in the Δ^9^-THC-d3 MRM channel was 45 area units, compared with a normal Δ^9^-THC-d3 response of approximately 850 area units at the internal-standard concentration routinely used in the method. Thus, the observed signal corresponded to approximately 5.3% of the normal internal-standard response under the applied analytical conditions. These findings demonstrated adequate selectivity and specificity of the method for the identification of the target analytes in hair samples.

#### 2.1.2. LOD and LLOQ

LOD and LLOQ were determined to assess the sensitivity of the method for each target analyte in hair. The evaluation was based on predefined signal-to-noise criteria with a signal-to-noise ratio of ≥3 for LOD and ≥10 for LLOQ. A preliminary instrumental assessment using multi-analyte standard solutions at 1 ng/μL was performed only to identify the approximate concentration range for subsequent matrix-based evaluation. Final method LOD and LLOQ values were established using matrix-matched hair samples through serial 1:2 dilutions starting from 25 and 50 pg/mg and extending to concentrations below the expected analytical limits. The experimentally tested concentration levels were used to identify the lowest concentrations fulfilling the predefined LOD and LLOQ criteria. The experimentally tested concentration levels corresponding to the LOD were 1.56 and 3.13 pg/mg, depending on the analyte. The selected LOD levels were confirmed by replicate determinations performed on three different analytical days (*n* = 3), with the predefined S/N ≥ 3 criterion consistently fulfilled. LLOQ values were established at the experimentally tested levels of 6.25 or 12.5 pg/mg, depending on the analyte. The selected LLOQ for each analyte was confirmed in five replicate analyses (*n* = 5) of matrix-matched samples prepared at the corresponding nominal concentration, with the predefined signal-to-noise criterion (S/N ≥ 10) consistently fulfilled. For analytes with an LLOQ of 6.25 pg/mg, this level was established on the basis of the predefined S/N ≥ 10 criterion; bias and precision were not independently determined at this concentration. Therefore, the 6.25 pg/mg value should be interpreted as an S/N-based LLOQ rather than as a fully validated lower calibration limit. Overall, LOD values ranged from 1.56 to 3.13 pg/mg and LLOQ values from 6.25 to 12.5 pg/mg (Table 1).

#### 2.1.3. Linearity

Linearity was evaluated using matrix-matched calibration curves prepared by standard addition using blank hair samples collected from the nuchal region of a subject with no reported history of drug use. Seven matrix-matched calibration levels were prepared at 0, 12.5, 25, 50, 100, 200, and 400 pg/mg, with the internal standards added at a constant concentration of 100 pg/mg. The zero level corresponded to the matrix blank and contained only the internal standards; it was used to verify the absence of relevant background signals and was excluded from the regression model. The lowest non-zero calibrator was 12.5 pg/mg. This concentration coincided with the analyte-specific LLOQ for analytes with an LLOQ of 12.5 pg/mg; lower analyte-specific LLOQ values were assessed independently as described in Section 2.1.2. Accordingly, the formally calibrated quantitative range was 12.5–400 pg/mg. For analytes with an S/N-based LLOQ of 6.25 pg/mg, concentrations between 6.25 and <12.5 pg/mg would fall below the validated calibration range. No authentic-sample result was affected by this limitation, as the lowest target-analyte concentration observed was 37.81 pg/mg. Calibration data were fitted using weighted least-squares linear regression with a 1/x weighting factor. Five independent calibration curves were prepared on five separate days, and each calibration level was analyzed in triplicate. The R^2^ values reported in Table 1 represent the mean ± standard deviation obtained from the five independent calibration curves and were ≥0.992 for all investigated analytes. Inspection of the residuals showed no systematic concentration-dependent trend. Back-calculated calibrator concentrations were considered acceptable when within ±15% of the corresponding nominal concentration, with ±20% allowed when the calibrator corresponded to the analyte-specific LLOQ. Across the five independent calibration runs, the observed back-calculated bias ranged from −13.72% to +13.92% across all analytes and non-zero calibration levels; therefore, all calibrators fulfilled the acceptance criterion. The upper limit of quantification (ULOQ) of the matrix-matched calibration curve was established at 400 pg/mg. Authentic samples producing analytical responses above the ULOQ were subjected to dilution and subsequent reanalysis to obtain a response within the validated calibration range. Final concentrations were calculated from the diluted measurement after application of the corresponding dilution factor.

#### 2.1.4. Accuracy and Precision

Accuracy and precision were evaluated at three quality control (QC) levels to assess the reliability of the developed method in hair. Low, medium, and high QC levels were analyzed in triplicate on five different days, resulting in fifteen measurements for each analyte at each concentration level. The low QC level was set at 30 pg/mg, above the LLOQ for all analytes, while the medium QC level was 80 pg/mg, representing approximately 20% of the upper concentration level of the matrix-matched calibration curve. The high QC level corresponded to approximately 75% of the highest calibration point. Accuracy was expressed as percentage error (ERR%) calculated by comparing the mean measured concentration with the corresponding nominal concentration. Precision was expressed as coefficient of variation (CV%). For each QC level, triplicate measurements were performed on five different analytical days. Within-day precision was calculated for each analytical day from the three replicate measurements, and the resulting five daily CV values were averaged to obtain the reported within-day CV. Between-day precision was calculated from the five daily mean concentrations as the standard deviation of the daily means divided by their overall mean and expressed as a percentage. A combined precision estimate was calculated by combining the within-day and between-day CV values according to the following equation: combined precision estimate = √[(CVwithin-day)^2^ + (CVbetween-day)^2^]. Acceptance criteria were set at CV values < 20% for the low QC level and <15% for the medium and high QC levels. Within-day precision ranged from 0.81% to 7.26%, between-day precision ranged from 0.76% to 11.98%, and the combined precision estimate ranged from 1.11% to 13.66%. Accuracy, expressed as percentage error (ERR%), ranged from −13.74% to +6.17%. All values met the predefined acceptance criteria and are summarized in Table 2.

#### 2.1.5. Matrix Effect, Recovery and Process Efficiency

Matrix effect (ME), recovery (RE), and process efficiency (PE) were evaluated according to the experimental design described by Matuszewski et al. [25]. The experiments were performed using blank hair from a single donor; therefore, the reported ME values represent absolute matrix-effect estimates for the matrix source used, whereas interindividual relative matrix-effect variability was not assessed. Three QC levels were investigated: low QC at 30 pg/mg, medium QC at 80 pg/mg, and high QC at 300 pg/mg. For each level, three sample sets were prepared: neat standard solutions, blank hair extracts fortified after extraction, and blank hair samples fortified before extraction.

ME was assessed by comparing the analytical response of post-extraction fortified samples with that of neat standard solutions. RE was calculated by comparing pre-extraction and post-extraction fortified samples, whereas PE was determined by comparing pre-extraction fortified samples with neat standard solutions, thereby accounting for the combined influence of extraction recovery and matrix-related ionization effects. Isotopically labeled internal standards were used to compensate for matrix-dependent variability. Five replicate determinations were performed on five different days for each analyte and QC level. For each analyte, the final value was reported as the mean of the three concentration levels. ME, RE, and PE were calculated separately at the low, medium, and high QC levels. For each analyte, the values reported in Table 2 represent the arithmetic mean of the results obtained across the three concentration levels. The complete level-specific ME, RE, and PE values, together with the corresponding coefficients of variation (CV%), are provided in Supplementary Table S1. Thus, mean PE was calculated as the arithmetic mean of the three level-specific PE values and not from the product of mean ME and mean RE. Although PE = (ME × RE)/100 at each matched QC level, mean PE is therefore not necessarily equal to (mean ME × mean RE)/100. When considered as analyte means across the three QC levels, ME values ranged from 88.65% to 109.93%, RE values from 85.91% to 110.88%, and PE values from 89.83% to 117.14% (Table 2). However, the level-specific values reported in Supplementary Table S1 showed wider ranges, namely 80.69–119.13% for ME, 81.77–130.12% for RE, and 77.80–129.67% for PE. The largest level-specific deviations included an RE of 130.12% and a PE of 124.20% for 9R-HHC at the high QC level, together with a PE of 77.80% at the low QC level. Thus, although the analyte-mean values were generally close to 100%, concentration-dependent deviations were observed for some analytes and should be considered when interpreting quantitative performance. A concentration-dependent pattern was also observed for ME, with values tending to increase from the low to the high QC level for most analytes. Because ME was calculated at each concentration level as the ratio between the response of post-extraction fortified matrix samples and that of the corresponding neat standards, this systematic trend may reflect concentration-dependent differences between these response sets and cannot be attributed exclusively to matrix-related ionization effects. The present experimental design does not allow the relative contribution of these factors to be distinguished; therefore, the level-specific ME values should be interpreted accordingly. Because the analytical panel included several ester-containing compounds, recovery data were additionally examined to assess the potential effect of the complete M3 treatment on these analytes. In the experimental design used for recovery determination, pre-extraction fortified samples were exposed to the complete sample-preparation procedure, including incubation with M3 reagent at 100 °C for 60 min, whereas post-extraction fortified samples were not exposed to this treatment.

Recovery values for the ester-containing analytes ranged from 85.91% to 107.65%. Specifically, recovery was 85.91% for 9R-HHC-O, 100.41% for 9R-HHC-P-O, 100.85% for Δ^8^-THC-P-OAc, 101.06% for MDMB-4en-PICA, 101.46% for 5F-EDMB-PICA, 98.66% for 5F-EMB-PICA, 106.54% for MDMB-4en-PINACA, 99.22% for EDMB-PINACA, 107.65% for 4F-MDMB-BUTINACA, and 103.43% for 4F-MDMB-BUTICA. These recovery results confirm that the parent ester-containing analytes remained analytically detectable following the complete M3 treatment; however, recovery values alone cannot exclude partial chemical transformation during sample preparation and should not be interpreted as a direct stoichiometric measure of conversion. For 9R-HHC-O, potential deacetylation to 9R-HHC was therefore additionally assessed using blank hair fortified exclusively with 9R-HHC-O, as described in Section 2.1.1. In this experiment, any potential 9R-HHC formation remained below the validated LOD, corresponding to an estimated upper-bound molar conversion of <1.2%.

**Table 2:** Accuracy, within-day precision, between-day precision, combined precision estimate, mean ME, RE and PE values obtained for the investigated analytes at the different quality control concentration levels. Values are reported to two decimal places for consistency and do not imply greater analytical precision than that demonstrated by the validated method.

Analyte	Between-Day Precision (%CV)			Within-Day Precision (%CV)			Accuracy (%ERR)			Combined Precision Estimate (%CV)			Mean ME (%)	MeanRE (%)	Mean PE (%)
	Low QC	Mid QC	High QC	Low QC	Mid QC	High QC	Low QC	Mid QC	High QC	Low QC	Mid QC	High QC			
9R-HHC	6.90	2.69	1.51	5.66	2.67	1.18	−0.37	−0.44	−1.23	8.92	3.79	1.92	88.65	110.88	99.08
9R-HHC-O	11.11	6.81	2.70	4.78	2.49	2.29	−2.23	−2.06	−1.82	12.09	7.25	3.54	109.76	85.91	94.28
9R-HHC-P	10.24	9.05	2.27	4.76	4.86	1.65	−0.14	2.64	0.99	11.29	10.27	2.81	100.98	100.60	100.19
9R-HHC-P-O	7.27	6.48	2.77	6.67	5.31	3.11	−5.11	3.37	−4.11	9.87	8.38	4.16	101.22	100.41	101.59
MDMB-4en-PICA	3.98	2.59	0.93	2.80	2.83	0.89	−2.79	0.20	−0.68	4.87	3.84	1.29	101.89	101.06	102.99
5F-Cumyl-PICA	6.68	2.50	0.90	4.85	2.20	0.95	1.27	0.39	0.14	8.25	3.33	1.31	104.03	92.52	96.20
5F-EDMB-PICA	5.15	2.89	1.09	2.74	1.62	1.12	0.94	0.24	−0.34	5.83	3.31	1.56	99.39	101.46	100.78
5F-EMB-PICA	5.04	2.01	1.05	3.45	2.02	1.15	−3.25	−1.69	−0.13	6.11	2.85	1.56	100.94	98.66	99.41
5F-APP-PICA	11.07	3.77	2.77	5.84	4.12	2.13	3.51	−2.71	−3.14	12.52	5.58	3.49	100.35	99.64	99.97
ADB-4en-PINACA	10.14	9.03	1.80	5.47	4.98	1.10	−13.74	−4.50	−0.68	11.52	10.31	2.11	89.97	108.28	97.49
MDMB-4en-PINACA	8.89	6.95	3.46	5.10	4.67	3.21	−2.80	1.24	−2.72	10.25	8.37	4.72	109.93	106.54	117.14
EDMB-PINACA	8.89	7.06	3.88	5.28	4.86	3.80	1.02	1.89	−1.68	10.34	8.57	5.43	100.62	99.22	98.85
5Cl-AB-PINACA	6.88	4.47	5.34	7.11	3.81	4.17	−4.78	−1.79	2.44	9.89	5.87	6.78	103.39	99.61	100.34
5F-APP-PINACA	7.84	2.53	7.88	6.24	1.97	5.70	−3.98	−7.22	2.12	10.02	3.21	9.73	100.04	89.84	89.83
4F-MDMB-BUTINACA	8.49	11.98	4.59	5.94	6.56	4.14	1.18	2.26	−2.22	10.36	13.66	6.18	94.36	107.65	101.75
ADB-BUTINACA	4.64	6.09	2.31	2.64	3.66	1.72	−8.22	−4.18	−5.87	5.34	7.11	2.88	93.17	106.84	99.62
Δ^8^-THC	4.77	4.11	5.94	5.52	2.58	4.57	−6.88	−6.27	−1.46	7.30	4.85	7.49	102.77	102.92	105.80
Δ^8^-THC-P	8.22	4.86	7.30	6.28	3.61	6.11	−9.01	−2.21	−0.77	10.34	6.05	9.52	99.28	101.45	100.72
Δ^8^-THC-P-OAc	5.82	6.22	6.74	4.79	5.44	5.77	−6.24	−3.88	4.97	7.54	8.26	8.87	97.04	100.85	97.78
Δ^9^-THC-P	5.91	7.88	3.46	7.09	4.21	4.19	−4.99	6.17	−2.55	9.23	8.93	5.43	98.52	99.56	98.11
BZO-4en-POXIZID	8.94	9.25	3.81	3.00	4.10	3.88	−4.06	−2.11	−5.68	9.43	10.12	5.44	98.18	92.14	90.36
BZO-POXIZID	10.94	6.28	3.50	5.23	4.83	2.34	−3.07	3.39	−2.86	12.13	7.92	4.21	98.19	96.92	94.73
4F-MDMB-BUTICA	5.79	2.56	1.08	3.31	2.55	1.12	1.74	−0.30	−0.41	6.67	3.61	1.56	99.67	103.43	103.08
Cumyl-NBMINACA	5.38	2.93	1.57	5.03	2.61	1.65	−2.87	−1.40	−0.85	7.37	3.92	2.28	100.06	102.35	102.08
Cumyl-CH-MeGACLONE	4.71	3.99	1.19	3.92	1.42	1.21	−0.05	2.10	−0.39	6.13	4.24	1.70	99.83	102.99	102.81
ADB-5Br-INACA	7.66	3.67	0.76	7.26	3.50	0.81	−1.21	−0.69	−0.67	10.55	5.07	1.11	99.75	102.65	102.36

#### 2.1.6. Freeze–Thaw and Processed-Extract Stability

Freeze–thaw stability was evaluated at low, medium, and high QC levels over three freeze–thaw cycles. Although freeze–thaw conditions do not represent the routine storage conditions of intact hair samples, this experiment was retained as an additional assessment of analytical robustness. QC samples were stored at −20 °C for 24 h, thawed at room temperature, and analyzed after complete thawing; the cycle was repeated three times. Processed-extract stability was evaluated by maintaining prepared QC extracts in the autosampler at 10 °C for 24 h before reanalysis. Each prepared QC sample/extract was analyzed by three replicate injections; therefore, *n* = 3 refers to technical replicate injections and not to independently prepared replicate samples. The concentrations measured after the freeze–thaw treatment and after 24 h in the autosampler were compared with the corresponding nominal QC concentrations (30, 80, and 300 pg/mg). Freshly prepared T0 comparison samples were not analyzed contemporaneously; therefore, the percentage values reported in Table 3 represent deviations from the nominal concentrations rather than stability bias relative to T0. All measured concentrations showed deviations from the corresponding nominal values within ±15% under the tested conditions. Accordingly, these experiments should be interpreted as additional robustness assessments under the specified freeze–thaw and processed-extract conditions rather than as formal stability studies based on comparison with freshly prepared T0 samples. No consistent structure-dependent trend in stability was observed among the different cannabinoid classes under the tested conditions. In addition, the potential effect of the M3 sample-preparation conditions on ester-containing analytes was assessed through the pre-extraction/post-extraction fortification design used for recovery evaluation and through review of individually fortified samples, as described in Section 2.1.1 and Section 2.1.5. The stability of the prepared multi-analyte stock and working solutions was not experimentally assessed within the present validation study. Storage conditions and validity periods specified by the manufacturers applied to the certified reference materials and should not be interpreted as an experimental evaluation of the stability of the subsequently prepared multi-analyte solutions.

### 2.2. Application to Authentic Samples

The analytical method was retrospectively applied to authentic hair samples collected at the Forensic Toxicology Laboratory of the University of Rome Tor Vergata. The samples were obtained from drivers who underwent forensic toxicological testing following suspension of driving licenses for driving under the influence of alcohol and/or drugs. Between January 2022 and September 2025, *n* = 100 authentic samples were selected according to a predefined inclusion criterion: all samples had initially yielded non-negative screening results and were confirmed as positive for cannabinoids. The Δ^9^-THC concentrations used for sample selection and subsequent descriptive stratification were obtained from the laboratory’s previously validated routine UPLC-MS/MS hair-testing method. ^24^ Δ^9^-THC was not included among the 26 target analytes of the method developed in the present study, and its concentration data were used exclusively as pre-existing routine toxicological information. Δ^9^-THC concentrations were retained in ng/mg for descriptive reporting because of their higher concentration range, whereas the target analytes investigated in the present study were expressed in pg/mg to provide a more appropriate numerical scale for their substantially lower concentrations.

**Figure 2:**
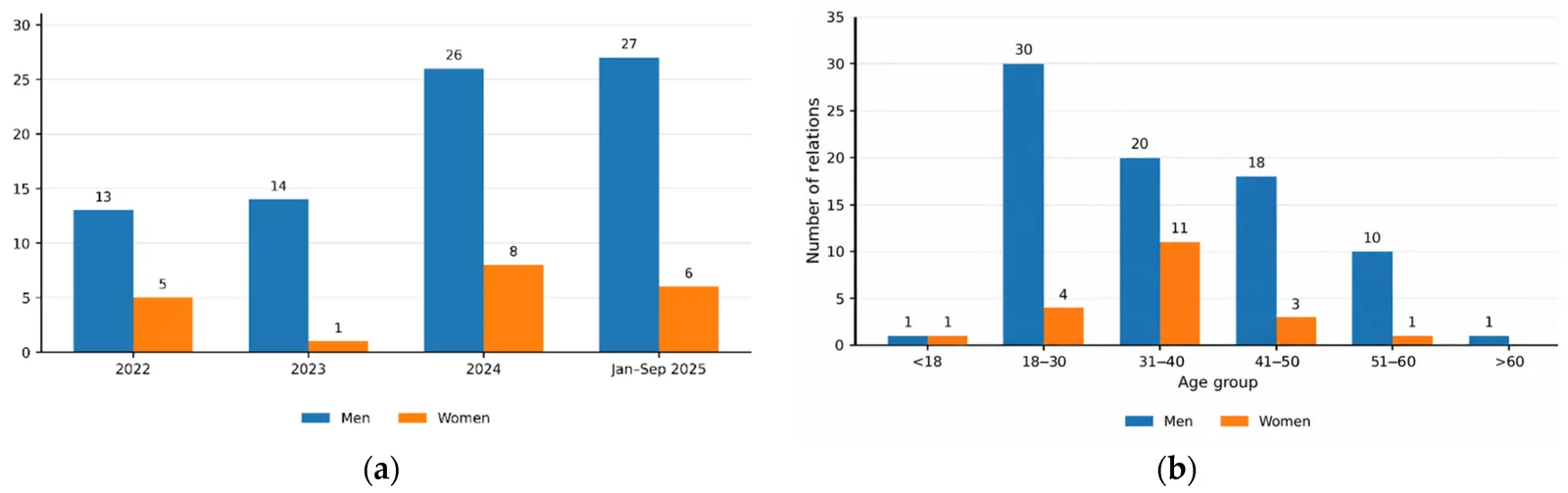
(**a**) Distribution of subjects by year and gender; (**b**) Distribution of subjects by gender and age group.

Basic descriptive statistical analysis was used to describe the main characteristics of the study population. Figure 2a shows the distribution of samples according to the reference year and the gender distribution.

Eighteen samples were collected in 2022, *n* = 15 in 2023, *n* = 34 in 2024, and *n* = 33 during the first nine months of 2025.

The study population comprised *n* = 80 males and *n* = 20 females. Figure 2a,b show the distribution of samples according to the reference year, together with the corresponding stratification by gender and age group.

Subjects aged 18–30 years and 31–40 years accounted for 65% of the total sample population. Twenty-one subjects belonged to the 41–50-year age group, *n* = 11 for the 51–60-year age group, *n* = 2 for subjects younger than 18 years, and *n* = 1 for people older than 60 years.

The mean age was 35.2 ± 11.3 years, with a minimum age of 15 years, a maximum age of 65 years and a mode of 22 years. Scalp hair was collected in *n* = 86 cases, whereas body hair was collected in *n* = 14 cases, comprising chest hair (*n* = 8), pubic hair (*n* = 5), and axillary hair (*n* = 1). For scalp hair, the 3 cm segment closest to the root was collected.

An additional classification was performed according to hair color. The categories brown and black were considered separately, whereas lighter shades, including blond, red, and white hair, were grouped into a single category. A separate category was maintained for subjects with gray or graying hair. Forty-one samples were classified as black, *n* = 37 as brown, *n* = 11 as gray or graying and *n* = 11 as light-colored. The Δ^9^-THC concentrations measured in the samples were categorized for descriptive purposes into four concentration ranges. The threshold values were established arbitrarily, solely for the purpose of a descriptive categorization of the sample set. In *n* = 43 cases, the Δ^9^-THC concentration was below 0.2 ng/mg; in *n* = 38 cases, between 0.21 and 0.5 ng/mg; in *n* = 13 samples, between 0.51 and 2 ng/mg; and above 2 ng/mg, in *n* = 6 cases.

**Figure 3:**
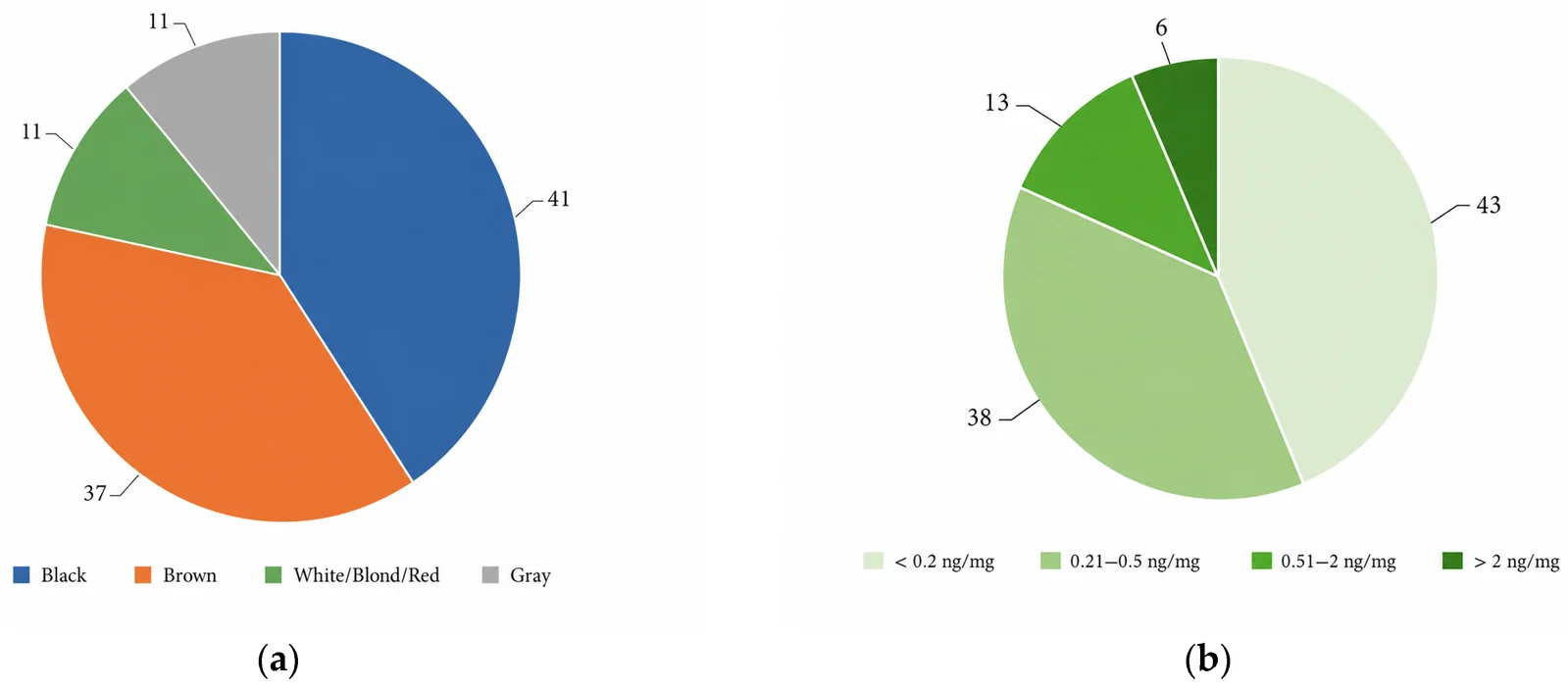
(**a**) Distribution of hair color categories. (**b**) Distribution of samples according to the Δ^9^-THC content detected in the hair matrix.

Figure 3 shows the classification of the hair matrices according to hair color (3a) and the distribution of samples according to the Δ^9^-THC content (3b).

### 2.3. Detection of Target Analytes in Authentic Samples

At least one of the *n* = 26 target analytes was detected in *n* = 23 samples, whereas no target analyte was detected in the remaining *n* = 77 samples. Among the *n* = 23 positive samples, *n* = 5 were collected in 2022, *n* = 4 in 2023, *n* = 8 in 2024, and *n* = 6 during the first nine months of 2025. Year-stratified positivity data were considered descriptive only and were not used to infer temporal trends.

Among the *n* = 23 positive subjects, *n* = 22 were males and *n* = 1 was female. The mean age of positive subjects was 36.0 ± 10.1 years, with a range of 20–58 years and a mode of 31 years. Out of the *n* = 23 positive cases, *n* = 18 involved scalp hair, *n* = 4 chest hair, and *n* = 1 axillary hair. No positive result was obtained from the *n* = 5 pubic hair samples analyzed.

When stratified by hair color, 10 of the 23 positive samples were collected from subjects with black hair, while seven positive samples were obtained from subjects with brown hair. Positive results were also observed in 3 of the 11 gray or graying hair samples and in 3 of the 11 light-colored hair samples.

Authentic samples producing analytical responses above the validated ULOQ of 400 pg/mg were diluted and subsequently reanalyzed to obtain an analytical response within the validated matrix-matched calibration range. The final concentrations reported for these samples were calculated from the concentrations measured after dilution and corrected for the corresponding dilution factor; therefore, they were not obtained by direct extrapolation of the calibration curve beyond its validated range.

The distribution of positive findings according to the initial Δ^9^-THC concentration showed that *n* = 8/13 positive samples (61.5%) belonged to the 0.51–2 ng/mg range. Six positive samples were observed among those with Δ^9^-THC concentrations above 2 ng/mg, representing all samples included in this category. 6/43 positive samples (14.0%) were identified among samples with Δ^9^-THC concentrations below 0.2 ng/mg, whereas *n* = 3/38 positive samples (7.9%) were observed in the 0.21–0.5 ng/mg range. These proportions are reported for descriptive characterization only, and no inferential association between Δ^9^-THC concentration category and target-analyte positivity was assessed.

**Figure 4:**
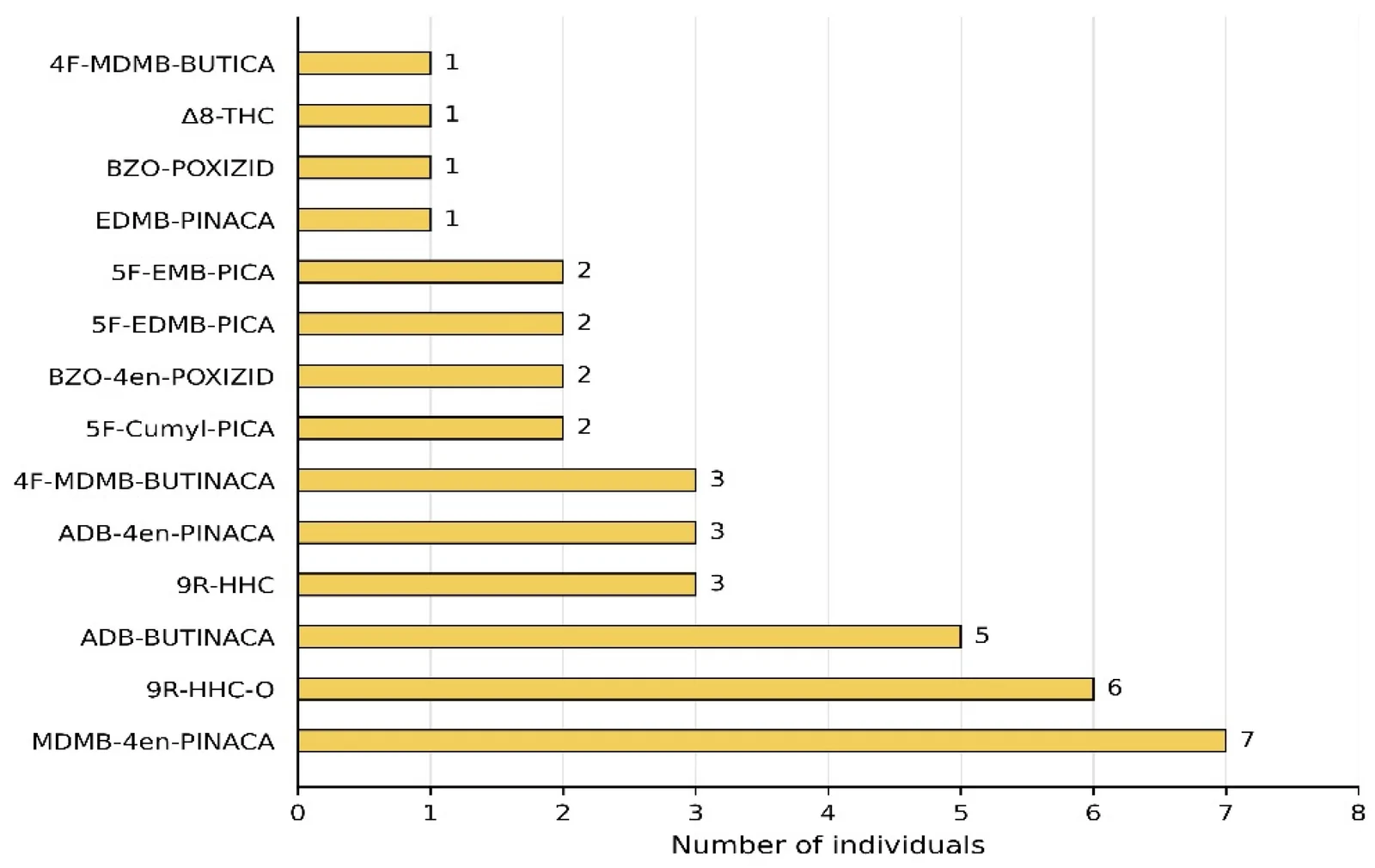
Number of analyte detections, stratified by analyte.

Overall, 39 analyte detections were identified across the *n* = 23 positive samples, corresponding to *n* = 14 different analytes (Figure 4). Among the 23 positive samples, eight contained one target analyte, 14 contained two target analytes, and one contained three target analytes; thus, 15/23 (65.2%) showed the co-detection of more than one target cannabinoid.

MDMB-4en-PINACA was the most frequently represented compound with *n* = 7 cases, followed by 9R-HHC-O (*n* = 6 cases) and ADB-BUTINACA (*n* = 5 cases).

9R-HHC, ADB-4en-PINACA, and 4F-MDMB-BUTINACA were each detected in three samples. 5F-Cumyl-PICA, BZO-4en-POXIZID, 5F-EDMB-PICA, and 5F-EMB-PICA were each detected in two samples. Single detections were observed for 4F-MDMB-BUTICA, Δ^8^-THC, BZO-POXIZID and EDMB-PINACA. The individual quantitative results for all 39 target-analyte detections, together with the corresponding anonymized sample identifier and matrix type, are reported in Table 4.

The single Δ^8^-THC-positive sample also contained Δ^9^-THC at 0.44 ng/mg. Re-examination of the chromatographic data showed two distinct peaks within the same injection at 7.35 and 7.50 min in both monitored transitions (*m*/*z* 315.2 → 193.1 and 315.2 → 123.0). The later peak differed by only 0.01 min from the Δ^8^-THC reference-standard retention time of 7.49 min and was therefore retained as a Δ^8^-THC finding. The concentrations of the detected analytes showed substantial inter-sample and inter-analyte variability (Table 4). For analytes detected in multiple samples, the arithmetic mean concentrations were 1256.38 pg/mg for MDMB-4en-PINACA (*n* = 7), 50.97 pg/mg for 9R-HHC-O (*n* = 6), 1103.69 pg/mg for ADB-BUTINACA (*n* = 5), 604.17 pg/mg for 9R-HHC (*n* = 3), 816.44 pg/mg for ADB-4en-PINACA (*n* = 3), 934.06 pg/mg for 4F-MDMB-BUTINACA (*n* = 3), 425.33 pg/mg for 5F-Cumyl-PICA (*n* = 2), 885.37 pg/mg for BZO-4en-POXIZID (*n* = 2), 563.04 pg/mg for 5F-EDMB-PICA (*n* = 2), and 1102.31 pg/mg for 5F-EMB-PICA (*n* = 2). For analytes detected in a single sample, the individual concentrations were 575.70 pg/mg for 4F-MDMB-BUTICA, 873.91 pg/mg for Δ^8^-THC, 736.32 pg/mg for BZO-POXIZID, and 1217.06 pg/mg for EDMB-PINACA. Individual results are provided in Table 4 to allow direct evaluation of the observed concentration distributions.

## 3. Discussion

The profile of compounds identified in the *n* = 100 authentic hair samples analyzed with the validated method was broadly consistent with substances reported by international, European, and Italian monitoring systems, supporting the relevance of the results in relation to the current landscape of synthetic cannabinoids. In particular, MDMB-4en-PINACA, 9R-HHC-O, and ADB-BUTINACA were among the most frequently detected analytes, reflecting the evolution documented by major international studies and European early warning systems [30,31]. The present method was not intended to maximize panel size or to outperform previously published approaches in all analytical parameters, but rather to provide a sensitive and routinely applicable targeted assay including several contemporary synthetic and semi-synthetic cannabinoids of current forensic relevance. In terms of analytical sensitivity, Liying et al. [14] reported LODs of 0.5–5 pg/mg and LLOQs of 1–10 pg/mg for a panel of 29 synthetic cannabinoids and metabolites, whereas the present method showed LODs of 1.56–3.13 pg/mg and LLOQs of 6.25–12.5 pg/mg. Yang et al. [22], using a broader 65-analyte panel, reported LODs of 10–15 pg/mg and LOQs of 25–40 pg/mg. These comparisons should be interpreted cautiously because the investigated analytes, sample-preparation procedures, calibration strategies, and validation designs differ among studies.

Several studies in the literature report that the synthetic cannabinoid market has been dominated by specific structural families, particularly those characterized by MDMB-4en and ADB- substituents, which are known for their high potency and widespread occurrence in clinical and forensic cases [32]. The predominance of MDMB-4en-PINACA in the analyzed samples is consistent with global observations, as several studies identified it as one of the most frequently reported substances worldwide since 2019 [33].

ADB-BUTINACA and ADB-4en-PINACA were also detected in our study, in line with recent reports documenting their increasing occurrence [21,34]. Metabolites of synthetic cannabinoids have also been investigated and detected in human hair in previous studies [14], highlighting the potential value of metabolite monitoring in forensic hair analysis. However, metabolites were not included in the targeted analytical panel of the present study. The frequent co-detection of multiple target cannabinoids suggests that exposure to more than one cannabinoid was relatively common among positive cases. However, because hair provides a retrospective window of exposure, these findings cannot establish simultaneous consumption or distinguish between separate exposures and the use of products containing multiple cannabinoids. Moreover, the present targeted panel does not allow conclusions regarding multidrug use involving other drug classes. An additional descriptive observation was the higher proportion of target-analyte-positive samples in the upper Δ^9^-THC concentration categories. Target analytes were detected in 8/13 samples (61.5%) with Δ^9^-THC concentrations of 0.51–2 ng/mg and in 6/6 samples (100%) with Δ^9^-THC concentrations above 2 ng/mg, compared with 6/43 (14.0%) and 3/38 (7.9%) in the two lower Δ^9^-THC categories, respectively. However, the Δ^9^-THC concentration categories were established for descriptive purposes, and the number of samples in the higher concentration groups was limited. Therefore, this observation should be interpreted cautiously and does not, by itself, establish an association between Δ^9^-THC concentration and the presence of the target cannabinoids.

Other synthetic cannabinoids, including 5F-Cumyl-PICA, 5F-EDMB-PICA, 5F-EMB-PICA, and EDMB-PINACA, were detected less frequently, reflecting their current marginal position in the global drug market. As reported by Banister [35] and Berg [36], these molecules represented a dominant group up to 2017–2018, before being progressively replaced, at least in part, by newer structural series. Their detection in the present study nevertheless demonstrates that compounds belonging to earlier generations may continue to be encountered in authentic forensic samples.

The detection of semi-synthetic cannabinoids was also of particular interest. 9R-HHC-O was the second most frequently detected analyte, followed by 9R-HHC. The dedicated 9R-HHC-O fortification experiment showed that any 9R-HHC formation following the complete M3 treatment remained below the validated 9R-HHC LOD, corresponding to an estimated upper-bound molar conversion of <1.2%. This finding argues against substantial deacetylation of 9R-HHC-O during sample preparation and supports, although does not by itself definitively prove, the interpretation of 9R-HHC and 9R-HHC-O as distinct analytical findings in the authentic samples. The presence of these compounds is consistent with the increasing emergence of hexahydrocannabinol derivatives in Europe and with their increasing relevance in forensic toxicology [12,31,37]. Their inclusion in the analytical panel therefore extends the applicability of the method beyond conventional synthetic cannabinoids and reflects the rapidly changing composition of the cannabinoid market.

The compounds identified in the present study were broadly consistent with those reported in recent European monitoring data. MDMB-4en-PINACA was one of the most frequently reported synthetic cannabinoids in Europe between 2019 and 2021, with its presence continuing to be documented in subsequent years [31]. ADB-BUTINACA has also emerged as an important compound in recent reports, with an increasing number of detections since 2021 [13], replacing molecules that were less stable and less available in regulated markets. The detection of 4F-MDMB-BUTINACA and 4F-MDMB-BUTICA is also consistent with the emergence of related structural types following the 2021 Chinese regulatory restrictions on synthetic cannabinoid precursors [38].

The identification of BZO-4en-POXIZID and BZO-POXIZID is noteworthy because POXIZID compounds represent a relatively recent addition to the range of synthetic cannabinoid structures encountered in forensic toxicology. Although these compounds were detected in only a small number of samples in the present study, their identification supports the need to include emerging structural classes in analytical methods intended for forensic application.

The findings are also consistent with the Italian monitoring context. The 2025 Annual Report to the Italian Parliament has documented the presence of MDMB-4en-PINACA, ADB-BUTINACA, 4F-MDMB-BUTINACA, and other synthetic cannabinoids belonging to the hexahydrocannabinol class in Italy [37].

The concentrations measured in the authentic samples showed substantial differences among analytes. MDMB-4en-PINACA showed marked inter-sample variability, whereas ADB-BUTINACA was detected at relatively high concentrations and 9R-HHC-O at substantially lower concentrations. However, these differences should not be interpreted directly in terms of dose, frequency of use, or toxicological significance. Hair incorporation may vary according to analyte physicochemical properties, including lipophilicity, ionization, and affinity for melanin and keratin; however, analyte-specific binding was not investigated in the present study. Accordingly, the quantitative results obtained in the present study should primarily be considered as analytical findings indicative of exposure and should be interpreted within the overall forensic context.

The application of the method to authentic samples also demonstrates the value of hair analysis for retrospective investigation of exposure to emerging cannabinoids. Hair was selected because it provides a retrospective detection window complementary to blood and urine, which primarily reflect more recent exposure; direct comparison of analytical sensitivity or diagnostic reliability across these matrices was beyond the scope of the present study and would require paired specimens analyzed using matrix-specific validated methods. Unlike conventional screening approaches, which are often focused on a limited number of established substances, the targeted multi-analyte strategy used in the present study allowed the simultaneous investigation of several structurally different cannabinoid classes. This approach is particularly relevant in forensic toxicology, where the composition of the illicit drug market can change more rapidly than routine analytical panels can be updated.

## 4. Materials and Methods

### 4.1. Materials

5F-APP-PICA, 5Cl-AB-PINACA, and 5F-APP-PINACA were purchased from LGC Standards (Guildford, United Kingdom). The other 23 reference standardswere obtained from Comedical (Trento, Italy). For quantitative analysis, delta-9-tetrahydrocannabinol-d3 (Δ^9^-THC-d3) and cannabidiol-d3 (CBD-d3) were employed, both supplied by LGC Standards (Guildford, UK). Methanol and water of UPLC/MS grade were obtained from Biosolve BV (Valkenswaard, The Netherlands). Dichloromethane (Suprasolv for GC–MS, Supelco^®^) was purchased from Sigma-Aldrich (Milan, Italy). The aqueous mobile phase (A1), organic mobile phase (B2), and M3 extraction reagent were supplied by Comedical (Trento, Italy). Publicly available descriptions identify M3 as an acidic aqueous buffer; in a previously published application, a pH of 3.4 was reported. A1 and B2 are supplied as proprietary aqueous and organic mobile phases, respectively. M3, A1, and B2 were used as ready-to-use commercial reagents according to the manufacturer’s specifications and without further modification. Their complete proprietary formulations are not disclosed by the manufacturer. M3-based extraction procedures have previously been employed in validated UHPLC–MS/MS methods for the determination of drugs and new psychoactive substances in keratin matrices, including synthetic cannabinoids [39,40].

#### Target Analytes

**Figure 5:**
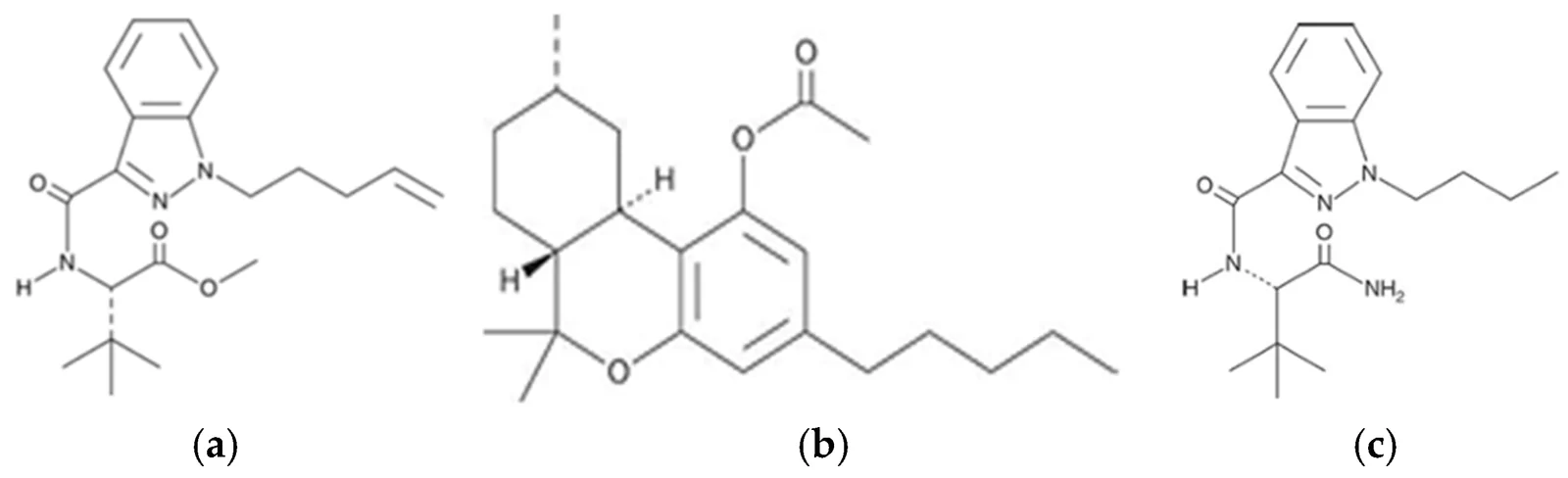
Chemical structures of the three most frequently detected target analytes in authentic hair samples: (**a**) MDMB-4en-PINACA, (**b**) 9R-HHC-O, (**c**) ADB-BUTINACA.

The analytical panel comprised 26 target analytes representing different synthetic, semi-synthetic, and related cannabinoids. The hexahydrocannabinol group included 9R-hexahydrocannabinol (9R-HHC), 9R-hexahydrocannabinol acetate (9R-HHC-O), 9R-hexahydrocannabiphorol (9R-HHC-P), and 9R-hexahydrocannabiphorol acetate (9R-HHC-P-O). A certified 9R-HHC reference standard was used for method development, validation, and chromatographic assignment. A certified 9S-HHC reference standard was not included in the original method-development and validation protocol. Consequently, the chromatographic behavior of 9S-HHC under the present analytical conditions was not experimentally characterized. Indole- and indazole-based synthetic cannabinoids included PICA derivatives MDMB-4en-PICA, 5F-Cumyl-PICA, 5F-EDMB-PICA, 5F-EMB-PICA, and 5F-APP-PICA, as well as PINACA derivatives ADB-4en-PINACA, MDMB-4en-PINACA, EDMB-PINACA, 5Cl-AB-PINACA, and 5F-APP-PINACA. The BUTINACA subclass included 4F-MDMB-BUTINACA and ADB-BUTINACA. Δ^8^-tetrahydrocannabinol (Δ^8^-THC) and related phytocannabinoid-derived or semi-synthetic analogues were also included: Δ^8^-tetrahydrocannabiphorol (Δ^8^-THC-P), Δ^8^-tetrahydrocannabiphorol acetate (Δ^8^-THC-P-OAc), and Δ^9^-tetrahydrocannabiphorol (Δ^9^-THC-P). The POXIZID subclass was represented by BZO-4en-POXIZID and BZO-POXIZID, two oxindole-based compounds characterized by a hydrazide linker; this structural feature may influence polarity, chemical reactivity, metabolism, and duration of action [41]. Additional emerging analytes were included based on their recent toxicological and forensic relevance: 4F-MDMB-BUTICA, Cumyl-NBMINACA, Cumyl-CH-MeGACLONE, and ADB-5Br-INACA. Δ^9^-THC-d3 and CBD-d3 were used as internal standards. The assignment of the two internal standards to individual target analytes was established empirically during method development on the basis of the overall analytical performance obtained for each analyte, including response stability and quantitative consistency, rather than solely on chromatographic retention-time proximity. The final internal-standard assignment for each analyte is reported in Table 5. Representative chemical structures of the three most frequently detected analytes in authentic samples are shown in Figure 5.

For ADB-5Br-INACA, only the ^81^Br isotopologue, corresponding to the precursor ion at *m*/*z* 355.1, was monitored in the validated MRM method. The ^79^Br isotopologue at *m*/*z* 353.1 was not included in the acquisition method; therefore, the ^79^Br/^81^Br isotopic ratio was not used as an additional identification criterion.

### 4.2. Sample Preparation

The sample preparation procedure was optimized to identify conditions suitable for the simultaneous analysis of all target compounds. Hair samples consisted primarily of scalp hair, typically 3–4 cm in length; chest, pubic, and axillary hair were also collected when available. Following collection, intact hair samples were stored dry, in the dark, at room temperature, in closed containers until analysis, under standardized conditions intended to minimize exposure to light, humidity, environmental contamination, and analyte degradation. These storage conditions are consistent with current recommendations for hair testing. The samples were finely cut using laboratory scissors until a homogeneous material, comparable to a fine powder, was obtained.

Approximately 25 mg of homogenized hair was accurately weighed and transferred into a screw-cap Pyrex glass tube. The decontamination step consisted of three consecutive washing cycles with 500 μL of dichloromethane. No aqueous washing step was included. The supernatants from the first two washing cycles were discarded, whereas the third wash fraction was collected and analyzed for all 100 authentic samples. Each third-wash fraction was transferred to an autosampler vial, fortified with the internal standards at the same concentration used for the corresponding hair-sample analysis, and directly injected into the UPLC-MS/MS system. No target analytes were detected in any of the 100 third-wash fractions. However, because the dichloromethane wash fractions were directly injected under chromatographic conditions beginning at 90% aqueous mobile phase, and because a wash-fraction-specific LOD was not experimentally established, these negative findings should be interpreted only as a qualitative control of the final washing step and have limited evidentiary value for excluding low-level analyte presence in the wash fractions. The samples were subsequently dried in a thermoblock at 100 °C for approximately 5 min to ensure complete solvent evaporation.

The dried samples were then treated with 500 μL of Comedical M3 reagent and 5 μL of internal standard solution. Δ^9^-THC-d3 and CBD-d3 were added at a concentration of 0.5 ng/μL. The tubes were centrifuged at 1800 rpm for 3 min and incubated in a thermoblock at 100 °C for 1 h, with the tubes kept closed, to allow digestion of the keratin matrix and extraction of the target analytes. The potential effect of these extraction conditions on ester-containing analytes was assessed during method validation by comparing samples fortified before and after extraction. Furthermore, in blank hair fortified exclusively with 9R-HHC-O at 300 pg/mg, the 9R-HHC response observed after the complete M3 treatment remained below the validated 9R-HHC LOD, corresponding to an estimated upper-bound molar conversion of <1.2%.

**Figure 6:**
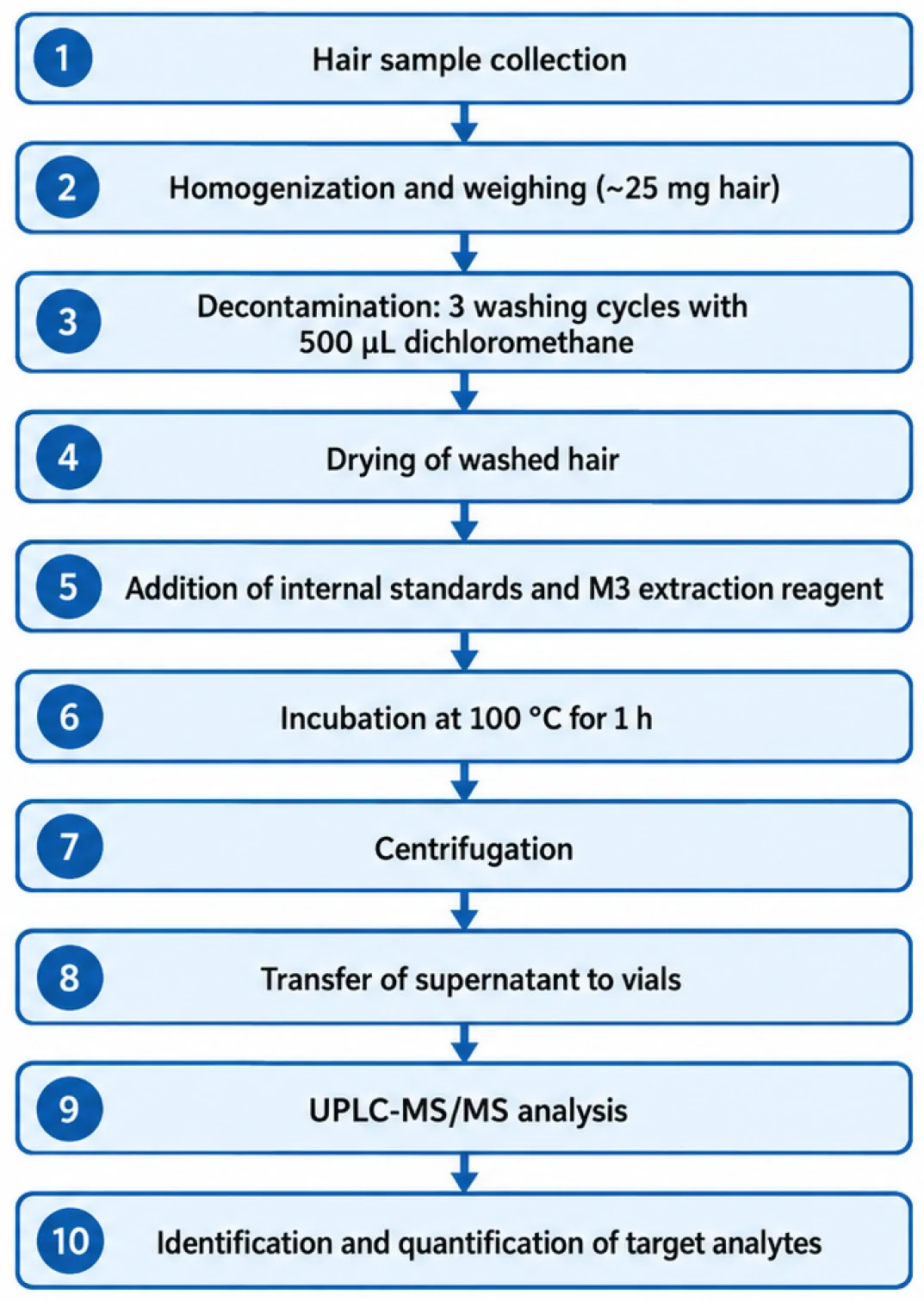
Schematic overview of the analytical workflow for hair-sample preparation and UPLC-MS/MS analysis of target analytes.

After incubation, the samples were cooled to room temperature and centrifuged again at 1800 rpm for 3 min. The resulting supernatants were transferred into 1.5 mL conical autosampler vials and injected into the UPLC-MS/MS system for qualitative and quantitative analysis. A schematic overview of the analytical workflow is provided in Figure 6. When the analytical response obtained from an authentic sample exceeded the upper limit of the validated matrix-matched calibration range (400 pg/mg), a 1:10 matrix-matched dilution was performed. Specifically, 50 µL of the processed authentic-sample extract was combined with 450 µL of a processed blank-hair matrix extract. Both extracts had been prepared according to the same sample-preparation procedure and contained the internal standards at the same concentration used in the analytical method. Therefore, the analyte concentration was reduced tenfold while the internal-standard concentration was maintained unchanged. The diluted extract was then reanalyzed, and the concentration measured within the validated calibration range was corrected by the corresponding dilution factor to obtain the final concentration reported for the authentic sample.

### 4.3. UPLC-MS/MS Analysis

Chromatographic separation was performed using an ACQUITY UPLC H-Class system (Waters, Milford, MA, USA). The detection was carried out in multiple reaction monitoring (MRM) mode using a Xevo TQD triple quadrupole mass spectrometer (Waters, Milford, MA, USA) equipped with an electrospray ionization source operated in positive mode (ESI+).

Chromatographic separation was performed using reversed-phase liquid chromatography on a Waters Atlantis Premier BEH C18 AX column (2.5 μm, 2.1 × 100 mm).

Following optimization, the Comedical Mobile Phase A1 aqueous phase and B2 organic phase were selected, based on their overall performance in terms of chromatographic resolution, analysis time, and compatibility with the electrospray ionization source.

The final chromatographic method employed binary gradient elution at a constant flow rate of 0.4 mL/min. The gradient started at 90% aqueous phase (A1) and decreased to 5% over 7 min, with intermediate conditions of 60% A1 at 3 min and 15% A1 at 5 min. The organic phase was then increased to 100% and maintained for approximately 1 min. The system was then returned to the initial conditions during the last 3 min for column re-equilibration.

The injection volume was set at 3 μL and the column temperature was maintained at 50 °C throughout the analysis. The total chromatographic run time was 11 min. An automatic needle/autosampler wash using water–methanol (50:50, *v*/*v*) was performed between consecutive injections. During the analysis of authentic samples, blank injections were included periodically within the analytical sequence and immediately after samples producing responses above the validated ULOQ to monitor potential carry-over. Mass spectrometric conditions were optimized for the simultaneous determination of the target analytes. The final source conditions were as follows: desolvation temperature, 450 °C; desolvation gas flow, 1000 L/h; cone gas flow, 20 L/h and capillary voltage, 2.5 kV. Cone voltage was optimized individually for each analyte, as reported in Table 5.

MRM transitions were optimized individually for each analyte to achieve adequate sensitivity, selectivity, and signal stability. Individual reference standards were initially infused into the mass spectrometer to optimize the instrumental parameters and identify the most suitable precursor ions and product ions.

The optimization workflow included initial full-scan mode acquisition to identify precursor ions, followed by product ion scan experiments to characterize fragmentation patterns. Precursor-ion scan analysis was subsequently used to further confirm the origin of the product ions. The most suitable transitions for qualitative and quantitative purposes were then selected for MRM acquisition.

The optimized precursor and product ions, collision energies, cone voltages, retention times, and acquisition windows for each analyte are reported in Table 5. Positive findings in authentic samples were accepted when the retention time was within ±2% of that of the corresponding reference standard, the selected MRM transitions were detected, the qualifier/quantifier ion ratio was within ±20% of the reference value, and the measured concentration was at or above the analyte-specific LLOQ. For isomeric compounds sharing identical MRM transitions and having overlapping ±2% retention-time acceptance windows, the retention-time tolerance was not used as a stand-alone identification criterion. Compound assignment additionally required a discrete chromatographic peak consistent with the retention time of the corresponding reference standard and chromatographically distinguishable from the potentially interfering isomer when both signals were present in the same injection. This additional evaluation was applicable to the authentic Δ^8^-THC finding, for which separate peaks corresponding to Δ^9^-THC and Δ^8^-THC were observed at 7.35 and 7.50 min, respectively, in both monitored transitions. For Δ^9^-THC-P and Δ^8^-THC-P, chromatographic resolution under simultaneous conditions was not experimentally established because a co-injection experiment was not performed; therefore, the ±2% retention-time criterion alone cannot be considered sufficient for unequivocal discrimination between these two isomers. For ADB-5Br-INACA, identification was based on retention time and the monitored MRM transitions of the *m*/*z* 355.1 precursor ion; confirmation based on the bromine isotopic ratio was not performed.

### 4.4. Method Validation

The analytical method was validated according to established forensic toxicology validation principles. The validation protocol included selectivity and specificity, calibration model and linearity, limits of detection and quantification, accuracy, within-day precision, between-day precision and combined precision estimate, matrix effect, recovery, process efficiency and carry-over, together with additional robustness assessments under freeze–thaw and processed-extract conditions. The experimental design and acceptance criteria applied to each validation parameter are described in the corresponding validation subsections, and the resulting analytical performance is reported in Section 2.1.

## 5. Limitations

This study has several limitations. First, its retrospective design and the selected nature of the sample population limit the generalizability of the findings. All hair samples had previously tested positive for cannabinoids in a forensic toxicology setting; therefore, the frequency of synthetic and semi-synthetic cannabinoid detection cannot be considered representative of the general population or of all drivers undergoing medico-legal assessment. In addition, the sample size was limited, and samples collected in 2025 covered only the first nine months of the year. Although all archived hair samples were stored under standardized dry, dark, room-temperature conditions consistent with hair-testing recommendations, analyte-specific long-term stability over the entire retrospective storage interval was not independently established. In addition, the freeze–thaw and processed-extract assessments were based on comparison with nominal QC concentrations rather than with contemporaneously prepared T0 samples, and the stability of the prepared multi-analyte stock and working solutions was not independently evaluated. Therefore, differences in positivity rates between collection years should be considered descriptive and should not be interpreted as evidence of temporal trends. Second, only two isotopically labeled internal standards were used for a structurally heterogeneous analytical panel of 26 analytes. Although validation data showed acceptable quantitative performance for all analytes, the use of a larger number of analyte-specific isotopically labeled internal standards could provide more comprehensive compensation for analyte-dependent variability. In addition, the observed contribution of native Δ^9^-THC to the Δ^9^-THC-d3 MRM channel, corresponding to approximately 5.3% of the normal internal-standard response under the tested conditions, may result in a positive bias in the Δ^9^-THC-d3 response and consequently in a negative bias in the calculated concentrations of analytes quantified using this internal standard. This effect may be more relevant in samples containing higher Δ^9^-THC concentrations. Matrix-effect experiments were performed using blank hair from a single donor; therefore, the reported matrix-effect values represent the matrix source used for validation, and interindividual relative matrix-effect variability across different hair sources was not assessed.

Another limitation concerns the heterogeneity of the keratin matrices analyzed. Although most samples consisted of scalp hair, a small number of body hair samples were also included. Differences in growth rate, drug incorporation, cosmetic treatment, and potential external contamination may affect the interpretation of analytical findings. Despite the use of a decontamination procedure, hair analysis cannot always completely distinguish systemic exposure from external contamination, particularly when analyte concentrations are low. In addition, because the decontamination protocol did not include an aqueous washing step and the third-wash analysis was qualitative only, external contamination cannot be definitively excluded. Although authentic samples producing responses above the validated ULOQ were diluted and reanalyzed within the calibration range, a dedicated dilution-integrity experiment using independently prepared quality-control samples above the ULOQ was not included in the original validation protocol; therefore, dilution integrity was not formally established. Selectivity was assessed using blank hair from a single donor and a limited interference panel; therefore, interindividual variability and interference from untested medication classes were not fully evaluated. A dedicated paired t = 0 versus t = 60 min stability experiment across the M3 treatment was not performed; therefore, minor transformation of ester-containing analytes during sample preparation cannot be completely excluded. Although the authentic Δ^8^-THC-positive sample showed two distinct chromatographic peaks corresponding to Δ^9^-THC and Δ^8^-THC in both monitored transitions, a dedicated co-injection of certified Δ^8^-THC and Δ^9^-THC reference standards at the highest authentic Δ^9^-THC concentration and a formal calculation of chromatographic resolution (Rs) were not performed. Therefore, the Δ^8^-THC assignment is supported by the same-injection chromatographic pattern and agreement with the individual reference-standard retention time, but a formal Rs value cannot be provided. Similarly, chromatographic resolution between Δ^9^-THC-P and Δ^8^-THC-P was not established by co-injection. A further limitation concerns the stereochemical identification of HHC. The method was developed and validated using a certified 9R-HHC reference standard only, whereas 9S-HHC was not included in the validation. Therefore, the chromatographic behavior of 9S-HHC under the present analytical conditions was not experimentally established. Because the two HHC epimers share the same nominal precursor/product-ion transitions, MRM detection alone cannot distinguish 9R-HHC from 9S-HHC. Although HHC peaks in authentic samples were assigned by comparison with the retention time of the available 9R-HHC reference standard, unequivocal epimer-specific identification cannot be ensured in the absence of experimental characterization and chromatographic resolution of 9S-HHC. Definitive discrimination would require both epimer reference standards and chromatographic conditions demonstrated to resolve them, potentially using a stereoselective analytical approach. Accordingly, the HHC findings should be interpreted as consistent with the 9R-HHC reference standard rather than as definitive exclusion of a possible 9S-HHC contribution. Finally, the method was developed and validated as a targeted approach covering 26 selected analytes. Given the rapid evolution of the synthetic cannabinoid market, newly emerging compounds not included in the panel may remain undetected. Moreover, the interpretation of hair concentrations is limited by the lack of well-established reference ranges and toxicological cutoffs for many of these compounds. Quantitative findings should therefore be interpreted cautiously and in conjunction with the available case information and broader forensic and clinical evidence.

## 6. Conclusions

The present study describes the development and validation of a sensitive UPLC-MS/MS method for the simultaneous detection and quantification of 26 synthetic, semi-synthetic and related cannabinoids, including hexahydrocannabinols, PICA, PINACA, BUTINACA, Δ^8^-THC-related compounds, POXIZID, and selected emerging analytes. The method demonstrated satisfactory overall analytical performance, with low pg/mg limits of detection and quantification, acceptable results for linearity, accuracy, and precision, and generally consistent mean matrix effect, recovery, and process efficiency, although some level-specific variability was observed, together with satisfactory robustness under the tested freeze–thaw and processed-extract conditions. Application of the validated method to *n* = 100 authentic forensic hair samples resulted in the detection of *n* = 23 positive cases, with *n* = 39 analyte detections corresponding to 14 different analytes. The compounds identified belonged to several structural families, including PINACA, BUTINACA, PICA, POXIZID, and hexahydrocannabinol derivatives. The observed profile was broadly consistent with recent international, European, and Italian monitoring data.

These findings demonstrate the applicability of the developed method to forensic hair analysis and highlight the importance of multi-analyte analytical strategies capable of encompassing both established synthetic cannabinoids and newly emerging semi-synthetic derivatives.

Our study provides an updated overview of synthetic cannabinoid occurrence in the studied population, supporting future epidemiological investigations and the development of more effective prevention and monitoring strategies for new psychoactive substances.

## Figures and Tables

**Table 1:** LOD, LLOQ, and R^2^ values for the investigated analytes. R^2^ values are reported as mean ± standard deviation from five independent calibration curves obtained using 1/x-weighted linear regression.

Analyte	LOD (pg/mg)	LLOQ (pg/mg)	R^2^
9R-HHC	3.13	12.5	0.994 ± 0.002
9R-HHC-O	1.56	6.25	0.996 ± 0.003
9R-HHC-P	3.13	12.5	0.995 ± 0.001
9R-HHC-P-O	3.13	12.5	0.994 ± 0.001
MDMB-4en-PICA	1.56	6.25	0.997 ± 0.003
5F-Cumyl-PICA	3.13	12.5	0.997 ± 0.002
5F-EDMB-PICA	1.56	6.25	0.998 ± 0.001
5F-EMB-PICA	1.56	6.25	0.997 ± 0.001
5F-APP-PICA	1.56	6.25	0.994 ± 0.002
ADB-4en-PINACA	1.56	6.25	0.996 ± 0.001
MDMB-4en-PINACA	1.56	6.25	0.995 ± 0.003
EDMB-PINACA	1.56	6.25	0.996 ± 0.002
5Cl-AB-PINACA	1.56	6.25	0.994 ± 0.001
5F-APP-PINACA	1.56	6.25	0.994 ± 0.001
4F-MDMB-BUTINACA	3.13	12.5	0.995 ± 0.001
ADB-BUTINACA	1.56	6.25	0.995 ± 0.002
Δ^8^-THC	3.13	12.5	0.993 ± 0.001
Δ^8^-THC-P	3.13	12.5	0.995 ± 0.002
Δ^8^-THC-P-OAc	3.13	12.5	0.994 ± 0.001
Δ^9^-THC-P	3.13	12.5	0.996 ± 0.003
BZO-4en-POXIZID	1.56	6.25	0.995 ± 0.001
BZO-POXIZID	1.56	6.25	0.994 ± 0.002
4F-MDMB-BUTICA	1.56	6.25	0.997 ± 0.001
Cumyl-NBMINACA	1.56	6.25	0.997 ± 0.003
Cumyl-CH-MeGACLONE	1.56	6.25	0.997 ± 0.003
ADB-5Br-INACA	3.13	12.5	0.994 ± 0.002

**Table 3:** Results of the freeze–thaw and processed-extract robustness assessments in hair matrix. Concentrations are reported as the mean of three technical replicate injections (*n* = 3) of the same prepared sample/extract. Percentage deviations were calculated relative to the nominal QC concentrations (30, 80, and 300 pg/mg), as freshly prepared T0 comparison samples were not analyzed. The predefined acceptance criterion for deviation from the nominal concentration was ±15%.

Analyte	Mean Freeze/Thaw Conc (pg/mg)			Autosampler Conc (pg/mg)			Deviation from Nominal fr/th (%)			Deviation from Nominal Aut (%)		
	Low QC	Mid QC	High QC	Low QC	Mid QC	High QC	Low QC	Mid QC	High QC	Low QC	Mid QC	High QC
9R-HHC	28.4	78.6	294.7	29.3	79.5	298.9	−5.33	−1.75	−1.77	−2.33	−0.62	−0.37
9R-HHC-O	31.2	81.2	302.4	30.7	80.0	304.2	4.00	1.50	0.80	2.33	0	1.4
9R-HHC-P	29.1	79.4	298.9	31.1	81.7	301.1	−3.00	−0.75	−0.37	3.67	2.13	0.37
9R-HHC-P-O	30.8	80.7	305.1	28.9	78.7	299.4	2.67	0.88	1.70	−3.67	−1.62	−0.20
MDMB-4en-PICA	32.0	82.1	291.8	30.2	82.4	297.2	6.67	2.62	−2.73	0.67	3.00	−0.93
5F-Cumyl-PICA	27.9	77.9	306.5	29.6	77.8	294.7	−7.00	−2.62	2.17	−1.33	−2.75	−1.77
5F-EDMB-PICA	30.3	80.3	303.9	31.4	79.8	298.6	1.00	0.37	1.30	4.67	−0.25	−0.47
5F-EMB-PICA	31.5	81.5	308.2	30.5	80.5	291.4	5.00	1.88	2.73	1.67	0.62	−2.87
5F-APP-PICA	29.7	79.7	297.6	28.7	79.3	307.1	−1.00	−0.37	−0.80	−4.33	−0.88	2.37
ADB-4en-PINACA	28.8	80.6	295.7	29.9	82.2	302.6	−4.00	0.75	−1.43	−0.33	2.75	0.87
MDMB-4en-PINACA	30.6	78.8	293.9	30.8	81.3	304.7	2.00	−1.50	−2.03	2.67	1.62	1.57
EDMB-PINACA	32.3	83.3	296.8	31.0	81.6	301.1	7.67	4.12	−1.07	3.33	2.00	0.37
5Cl-AB-PINACA	29.4	79.2	302.9	29.2	79.6	297.8	−2.00	−1.00	0.97	−2.67	−0.50	−0.73
5F-APP-PINACA	31.0	82.1	295.3	30.1	82.5	294.5	3.33	2.62	−1.57	0.33	3.12	−1.83
4F-MDMB-BUTINACA	30.1	78.4	304.8	28.8	77.7	296.5	0.33	−2.00	1.60	−4.00	−2.87	−1.17
ADB-BUTINACA	28.6	81.8	303.2	31.3	79.0	301.8	−4.67	2.25	1.07	4.33	−1.25	0.60
Δ^8^-THC	31.8	79.7	290.1	30.4	80.6	302.9	6.00	−0.37	−3.30	1.33	0.75	0.97
Δ^8^-THC-P	29.9	80.4	290.9	29.7	79.9	300.8	−0.33	0.50	−3.03	−1.00	−0.12	0.27
Δ^8^-THC-P-OAc	30.7	77.4	299.7	30.9	82.1	301.8	2.33	−3.25	−0.10	3.00	2.62	0.60
Δ^9^-THC-P	27.6	83.4	294.0	28.6	78.3	301.1	−8.00	4.25	−2.00	−4.67	−2.13	0.37
BZO-4en-POXIZID	32.1	82.7	298.6	31.2	80.2	299.1	7.00	3.38	−0.47	4.00	0.25	−0.30
BZO-POXIZID	29.2	83.0	304.7	29.5	78.5	290.9	−2.67	3.75	1.57	−1.67	−1.88	−3.03
4F-MDMB-BUTICA	28.9	78.4	300.7	30.6	77.9	297.2	−3.67	−2.00	0.23	2.00	−2.62	−0.93
Cumyl-NBMINACA	30.4	79.5	296.1	29.0	80.7	293.6	1.33	−0.62	−1.30	−3.33	0.88	−2.13
Cumyl-CH-MeGACLONE	31.3	77.9	292.2	30.3	83.1	294.7	4.33	−2.62	−2.60	1.00	3.87	−1.77
ADB-5Br-INACA	28.9	79.1	297.9	29.8	76.9	295.1	−3.67	−1.13	−0.70	−0.67	−3.87	−1.63

**Table 4:** Individual concentrations of target analytes detected in the 23 positive authentic samples. Each row represents one analyte detection. Concentrations are expressed as pg/mg. Where dilution and reanalysis were required because the analytical response exceeded the validated ULOQ, the final concentration reported includes application of the corresponding dilution factor. Values are reported to two decimal places for consistency and do not imply greater analytical precision than that demonstrated by the validated method.

Sample ID	Matrix Type	Analyte	Conc (pg/mg)
S01	Scalp hair	9R-HHC	434.47
S01	Scalp hair	9R-HHC-O	61.86
S02	Scalp hair	ADB-4en-PINACA	610.64
S02	Scalp hair	5F-Cumyl-PICA	285.20
S03	Scalp hair	9R-HHC-O	38.83
S04	Scalp hair	9R-HHC	343.69
S05	Chest hair	MDMB-4en-PINACA	64.89
S06	Scalp hair	ADB-BUTINACA	1026.30
S06	Scalp hair	MDMB-4en-PINACA	1716.63
S07	Chest hair	BZO-4en-POXIZID	614.02
S07	Chest hair	ADB-BUTINACA	807.42
S08	Scalp hair	9R-HHC-O	56.69
S09	Scalp hair	MDMB-4en-PINACA	1284.73
S09	Scalp hair	5F-EDMB-PICA	409.59
S10	Scalp hair	9R-HHC-O	52.39
S11	Scalp hair	9R-HHC-O	58.25
S12	Scalp hair	9R-HHC-O	37.81
S13	Chest hair	ADB-BUTINACA	943.93
S13	Chest hair	4F-MDMB-BUTINACA	1061.57
S14	Scalp hair	ADB-4en-PINACA	820.33
S15	Chest hair	MDMB-4en-PINACA	1568.20
S15	Chest hair	5F-EMB-PICA	939.42
S16	Scalp hair	BZO-4en-POXIZID	1156.72
S16	Scalp hair	BZO-POXIZID	736.32
S17	Scalp hair	ADB-4en-PINACA	1018.34
S17	Scalp hair	MDMB-4en-PINACA	1365.46
S17	Scalp hair	5F-EMB-PICA	1265.20
S18	Axillary hair	ADB-BUTINACA	1725.40
S18	Axillary hair	4F-MDMB-BUTINACA	1007.22
S19	Scalp hair	ADB-BUTINACA	1015.40
S19	Scalp hair	MDMB-4en-PINACA	904.28
S20	Scalp hair	5F-Cumyl-PICA	565.45
S20	Scalp hair	5F-EDMB-PICA	716.48
S21	Scalp hair	MDMB-4en-PINACA	1890.50
S21	Scalp hair	EDMB-PINACA	1217.06
S22	Scalp hair	9R-HHC	1034.36
S22	Scalp hair	Δ^8^-THC	873.91
S23	Scalp hair	4F-MDMB-BUTICA	575.70
S23	Scalp hair	4F-MDMB-BUTINACA	733.39

**Table 5:** MRM acquisition mode parameters. Internal Standards (ISs): (1) Δ^9^-THC-d3; (2) CBD-d3.

Analyte	Parent (*m*/*z*)	Daughter (*m*/*z*)	Dwell (s)	Cone (V)	Collision (V)	RT (min)	WA (min)	IS
9R-HHC	317.2	193.1 *	0.009	35	22	7.57	7.3–8.1	(1)
	317.2	123.1	0.009	35	36			
	317.2	81.1	0.009	35	22			
9R-HHC-O	359.3	193.2 *	0.009	35	30	8.04	7.8–8.5	(1)
	359.3	123.1	0.009	35	44			
	359.3	81.1	0.009	35	32			
9R-HHC-P	345.3	221.2 *	0.009	35	24	8.00	7.7–8.5	(1)
	345.3	123.1	0.009	35	38			
	345.3	81.1	0.009	35	24			
9R-HHC-P-O	387.3	221.0 *	0.009	35	24	8.51	8.2–8.9	(2)
	387.3	81.1	0.009	35	30			
MDMB-4en-PICA	357.2	212.1 *	0.009	30	14	6.21	6.0–6.5	(1)
	357.2	144.0	0.009	30	38			
	357.2	116.05	0.009	30	50			
5F-Cumyl-PICA	367.2	249.1 *	0.009	30	12	6.04	5.8–6.3	(1)
	367.2	119.05	0.009	30	24			
	367.2	91.05	0.009	30	50			
5F-EDMB-PICA	391.2	232.1 *	0.009	25	14	6.16	5.9–6.7	(1)
	391.2	144.1	0.009	25	40			
	391.2	116.05	0.009	25	50			
5F-EMB-PICA	377.2	232.1 *	0.009	30	12	5.94	5.7–6.2	(1)
	377.2	144.1	0.009	30	40			
5F-APP-PICA	396.3	144.0 *	0.009	36	44	5.60	5.1–6.1	(2)
	396.3	232.0	0.009	36	26			
ADB-4en-PINACA	343.2	213.1 *	0.009	25	22	5.92	5.7–6.2	(1)
	343.2	171.1	0.009	25	38			
	343.2	145.05	0.009	25	46			
MDMB-4en-PINACA	358.2	298.15 *	0.009	30	14	6.50	6.2–6.7	(1)
	358.2	213.1	0.009	30	22			
	358.2	145.05	0.009	30	42			
EDMB-PINACA	374.2	300.2 *	0.009	25	14	6.86	6.6–7.4	(1)
	374.2	215.1	0.009	25	22			
	374.2	145.2	0.009	25	42			
5Cl-AB-PINACA	365.2	145.0 *	0.009	36	44	5.72	5.5–5.9	(2)
	365.2	249.0	0.009	36	24			
	365.2	116.05	0.009	36	50			
5F-APP-PINACA	397.3	233.0 *	0.009	26	22	5.64	5.1–6.1	(2)
	397.3	145.0	0.009	26	46			
4F-MDMB-BUTINACA	364.2	304.2 *	0.009	25	24	5.95	5.7–6.5	(1)
	364.2	219.1	0.009	25	40			
ADB-BUTINACA	331.2	201.1 *	0.009	25	24	5.88	5.6–6.4	(1)
	331.2	145.1	0.009	25	40			
∆^8^-THC	315.2	193.1 *	0.009	35	30	7.49	7.2–8.0	(1)
	315.2	123.0	0.009	35	30			
∆^8^-THC-P	343.2	123.1 *	0.009	35	30	7.98	7.5–8.3	(1)
	343.2	221.2	0.009	35	24			
∆^8^-THC-P-OAc	385.2	165.1 *	0.009	35	24	8.57	8.1–8.9	(2)
	385.2	263.2	0.009	35	24			
∆^9^-THC-P	343.2	123.1 *	0.009	35	30	7.92	7.5–8.3	(2)
	343.2	221.2	0.009	35	30			
BZO-4en-POXIZID	334.3	105.1 *	0.009	35	18	6.52	6.2–7.0	(1)
	334.3	77.1	0.009	35	44			
	334.3	51.0	0.009	35	50			
BZO-POXIZID	336.2	105.1 *	0.009	30	16	6.82	6.6–7.1	(1)
	336.2	77.1	0.009	30	40			
4F-MDMB-BUTICA	363.2	218.1 *	0.009	25	12	5.83	5.5–6.5	(1)
	363.2	144.1	0.009	25	40			
	363.2	116.05	0.009	25	50			
Cumyl-NBMINACA	388.3	270.2 *	0.009	35	10	7.30	7.1–7.6	(1)
	388.3	253.2	0.009	35	20			
	388.3	145.1	0.009	35	40			
Cumyl-CH-MeGACLONE	399.3	281.2 *	0.009	30	16	7.00	6.8–7.3	(1)
	399.3	119.2	0.009	30	24			
	399.3	185.1	0.009	30	44			
ADB-5Br-INACA	355.1	225.0 *	0.009	35	30	5.52	5.4–5.8	(1)
	355.1	116.0	0.009	35	30			
	355.1	86.0	0.009	35	28			
∆^9^-THC-d_3_	318.2	123.0 *	0.009	35	34	7.35	7.0–8.0	(1)
	318.2	196.1	0.009	35	22			
CBD-d_3_	318.3	196.2 *	0.009	40	22	6.63	6.3–7.4	(2)
	318.3	123.2	0.009	40	32			

* Quantifier transition.

## Data Availability

The original contributions presented in this study are included in the article/Supplementary Material. Further inquiries can be directed to the corresponding author(s).

## Supplementary Materials

The following supporting information can be downloaded at: https://www.mdpi.com/article/10.3390/molecules31213816/s1, Table S1: Level-specific matrix effect (ME), recovery (RE), and process efficiency (PE) values and corresponding coefficients of variation (CV%) for the 26 target analytes at the low (30 pg/mg), medium (80 pg/mg), and high (300 pg/mg) QC levels.
