# Supplementary material for: Development and Validation of a UPLC-MS/MS Method for the Determination of Emerging Synthetic, Semi-Synthetic and Related Cannabinoids in Human Hair
Source: Molecules. 2026 Oct 22;31(21):3816. doi: 10.3390/molecules31213816 (PMC13764793; doi:10.3390/molecules31213816)
Supplement: Supplementary file 1 [file molecules-31-03816-s001.zip › molecules-4552173-supplementary.pdf]

Supplementary Material

Table S1. Level-specific matrix effect (ME), recovery (RE), and process efficiency (PE) values and corresponding coefficients of variation (CV%) for the 26 target analytes at the low (30 pg/mg), medium (80 pg/mg), and high (300 pg/mg) QC levels.

Analyte	ME%			ME CV(%)			RE%			RE CV(%)			PE%			PE CV(%)		
	Low QC	Mid QC	High QC	Low QC	Mid QC	High QC	Low QC	Mid QC	High QC	Low QC	Mid QC	High QC	Low QC	Mid QC	High QC	Low QC	Mid QC	High QC
9R-HHC	82.33	88.17	95.45	2.34	2.74	3.42	94.50	108.02	130.12	2.38	2.50	3.04	77.80	95.24	124.20	3.40	3.97	4.65
9R-HHC-O	100.96	109.19	119.13	2.89	3.69	3.80	88.40	81.77	87.56	3.03	3.50	4.08	89.25	89.28	104.31	4.47	5.07	6.03
9R-HHC-P	85.59	114.60	102.75	3.50	4.05	4.60	114.86	85.91	101.03	4.20	4.70	5.20	98.31	98.45	103.81	5.52	6.28	7.03
9R-HHC-P-O	93.41	102.15	108.10	3.91	4.33	4.94	102.41	97.70	101.12	5.08	2.37	2.49	95.66	99.80	109.31	6.77	5.35	6.15
MDMB-4en-PICA	96.05	101.38	108.24	4.85	4.99	2.64	102.75	97.14	103.29	2.87	3.48	3.88	98.69	98.48	111.80	5.52	6.25	4.55
5F-Cumyl-PICA	95.63	104.23	112.23	5.09	2.64	3.04	95.43	88.26	93.87	3.53	3.95	4.58	91.26	91.99	105.35	6.33	4.62	5.76
5F-EDMB-PICA	89.51	99.61	109.05	2.47	3.11	3.58	103.65	98.83	101.90	4.61	5.09	2.36	92.78	98.44	111.12	5.37	5.94	4.43
5F-EMB-PICA	92.48	101.29	109.05	3.14	3.64	3.92	103.59	94.95	97.44	2.37	2.54	3.45	95.80	96.17	106.26	3.50	4.59	5.32
5F-APP-PICA	93.03	99.42	108.60	3.25	3.85	4.79	103.14	94.28	101.50	3.44	3.50	3.99	95.95	93.73	110.23	4.72	5.42	6.22
ADB-4en-PINACA	81.58	90.55	97.78	4.20	4.53	5.03	108.01	105.92	110.91	4.29	4.94	5.06	88.11	95.91	108.45	5.72	6.44	7.34
MDMB-4en-PINACA	99.96	111.05	118.78	4.86	5.07	2.31	107.79	102.66	109.17	4.93	2.34	2.87	107.75	114.00	129.67	7.10	5.81	3.83
EDMB-PINACA	88.46	101.10	112.30	5.15	2.40	2.95	111.37	99.75	86.54	2.70	3.20	3.70	98.52	100.85	97.18	5.89	4.03	4.77
5Cl-AB-PINACA	80.69	114.48	115.00	2.40	2.95	3.50	122.93	87.93	87.97	3.70	4.20	4.70	99.19	100.66	101.17	4.44	5.17	5.92
5F-APP-PINACA	91.81	99.80	108.51	3.21	3.64	4.20	92.37	86.53	90.62	4.64	5.00	2.27	84.80	86.36	98.33	5.80	6.45	4.92
4F-MDMB-BUTINACA	85.88	94.92	102.28	3.68	4.19	4.40	106.78	102.56	113.61	2.42	2.58	3.37	91.70	97.35	116.20	4.37	4.79	5.43
ADB-BUTINACA	85.51	92.52	101.48	4.25	4.72	5.03	108.25	101.70	110.57	3.31	3.43	4.28	92.56	94.09	112.21	5.38	5.84	6.56
Δ^8-THC	96.08	103.81	108.42	4.40	4.89	2.63	103.90	98.39	106.47	3.99	4.89	5.10	99.83	102.14	115.43	6.55	6.81	6.02
Δ^8-THC-P	90.92	100.15	106.77	5.02	2.60	2.76	103.36	96.82	104.17	5.03	2.34	2.96	93.97	96.97	111.22	7.33	3.47	4.25
Δ^8-THC-P-OAc	87.07	97.78	106.27	2.28	3.14	3.40	103.37	98.11	101.07	2.45	2.94	3.87	90.00	95.93	107.41	3.53	4.32	5.41
Δ^9-THC-P	90.34	98.31	106.91	3.15	3.27	4.30	101.01	95.91	101.76	3.90	3.97	4.42	91.25	94.29	108.79	4.90	5.34	6.20
BZO-4en-POXIZID	90.16	97.84	106.54	3.75	4.12	4.77	96.73	87.01	92.68	4.95	5.14	2.41	87.21	85.13	98.74	5.80	6.51	5.42
BZO-POXIZID	91.25	97.54	105.78	4.14	4.67	4.89	108.06	93.10	89.60	2.40	2.86	3.05	98.60	90.81	94.78	4.51	5.21	5.98
4F-MDMB-BUTICA	90.47	98.56	109.98	4.87	5.01	2.64	105.93	99.35	105.01	3.28	3.80	3.96	95.83	97.92	115.49	5.92	6.30	4.59
Cumyl-NBMINACA	92.23	99.66	108.29	4.92	2.50	2.88	110.94	97.86	98.25	3.96	4.83	5.01	102.32	97.53	106.39	7.00	5.58	6.21

Cumyl-CH-MeGACLONE	93.09	100.61	105.79	2.57	2.85	3.35	104.23	100.19	104.55	4.96	2.45	2.64	97.03	100.80	110.60	5.49	3.51	4.27
ADB-5Br-INACA	91.58	98.70	108.97	3.12	3.37	4.25	105.55	98.67	103.73	2.95	2.95	3.88	96.66	97.39	113.03	4.11	4.60	5.79
